# Crystal structure and Hirshfeld surface analysis of 2-(4-nitro­phen­yl)-2-oxoethyl benzoate

**DOI:** 10.1107/S2056989019013975

**Published:** 2019-10-22

**Authors:** S. N. Sheshadri, C. S. Chidan Kumar, S. Naveen, M. K. Veeraiah, Kakarla Raghava Reddy, Ismail Warad

**Affiliations:** a Department of Chemistry, GSSS Institute of Engineering and Technology for Women, Mysuru 570 016, Karnataka, India; b Department of Engineering Chemistry, Vidya Vikas Institute of Engineering & Technology, Visvesvaraya Technological University, Alanahally, Mysuru 570 028, Karnataka, India; c Department of Physics, School of Engineering and Technology, Jain University, Bangalore 562 112, India; d Department of Chemistry, Sri Siddhartha Institute of Technology, Tumkur 572 105, Karnataka, India; e School of Chemical & Biomolecular Engineering, The University of Sydney, Sydney, NSW, Australia; f Department of Chemistry, Science College, An-Najah National University, PO Box 7, Nablus, West Bank, Palestinian Territories

**Keywords:** crystal structure, R_{2}^{2}(16) ring motif, C-H⋯O hydrogen bonds, offset π–π inter­actions, Hirshfeld surface analysis

## Abstract

The title com­pound, C_15_H_11_NO_5_, is relatively planar, with the planes of the two aromatic rings being inclined to each other by 3.09 (5)°. In the crystal, mol­ecules are linked by a pair of C—H⋯O hydrogen bonds, forming inversion dimers, which enclose an *R*_2_^2^(16) ring motif. The dimers are linked by a further pair of C—H⋯O hydrogen-bonds forming ribbons enclosing *R*_4_^4^(26) ring motifs. The ribbons are linked by offset π–π inter­actions [centroid–centroid distances = 3.6754 (6)–3.7519 (6) Å] to form layers parallel to the *ac* plane. Through Hirshfeld surface analyses, the *d*_norm_ surfaces, electrostatic potential and two-dimensional fingerprint (FP) plots were examined to verify the contributions of the different inter­molecular contacts within the supra­molecular structure. The shape-index surface shows that two sides of the mol­ecule are involved with the same contacts in neighbouring mol­ecules, and the curvedness plot shows flat surface patches that are characteristic of planar stacking.

## Abstract 2

The title com­pound, 2-(4-nitro­phen­yl)-2-oxoethyl benzoate, is relatively planar with the two aromatic rings being inclined to each other by 3.09 (5)°.

## Chemical context   

Photoreleasable protecting groups have been of long-standing inter­est for their diverse applications in various multistep syntheses (Ruzicka *et al.*, 2002 ▸; Literák *et al.*, 2006 ▸). The reaction between an acid and a phenacyl bromide yields the keto ester derivative. As a protecting group, the ester derivatives are well known as protecting groups for carb­oxy­lic acids in chemical synthesis (Rather & Reid, 1919 ▸; Literák *et al.*, 2006 ▸). They can easily be cleaved under com­pletely neutral or mild conditions (Sheehan & Umezawa, 1973 ▸) and are therefore used for the identification of organic acids. Versatile applications of these com­pounds are seen in the field of synthetic chemistry, such as in the synthesis of oxazoles and imidazoles (Huang *et al.*, 1996 ▸), as well as benzoxazepine (Gandhi *et al.*, 1995 ▸), and they are also useful in peptide synthesis. Studies reveal an inhibitory activity against two isozymes of 11b-hy­droxy­steroid de­hydrogenases (11b-HSD1 and 11b-HSD2), which catalyze the interconversion of active cortisol and inactive cortisone (Zhang *et al.*, 2009 ▸). Researchers have reported the synthesis and photolysis studies of a number of phenacyl esters. The commercial importance of phenacyl benzoates arose due to their applications in various fields of chemistry. In continuation of our work on such mol­ecules (Kumar *et al.*, 2014 ▸; Chidan Kumar *et al.*, 2014 ▸), we report herein on the crystal and mol­ecular structure of 2-(4-nitro­phen­yl)-2-oxoethyl benzoate.
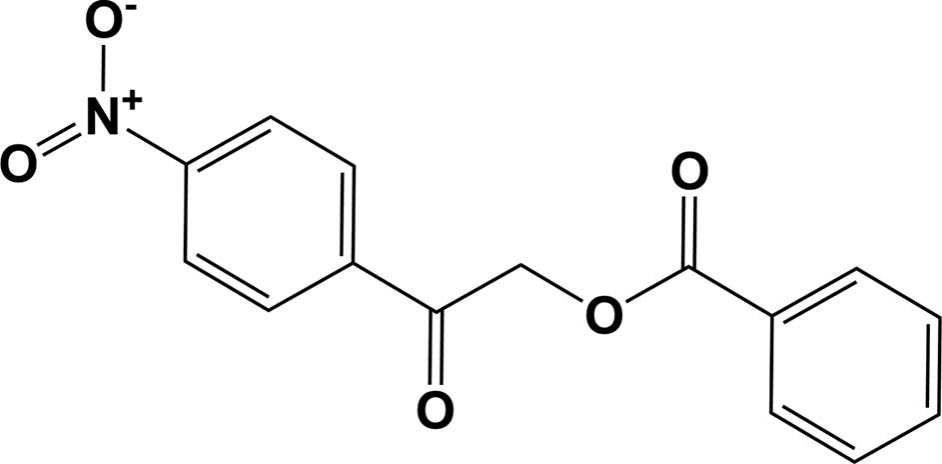


.

## Structural commentary   

**Figure 1:**
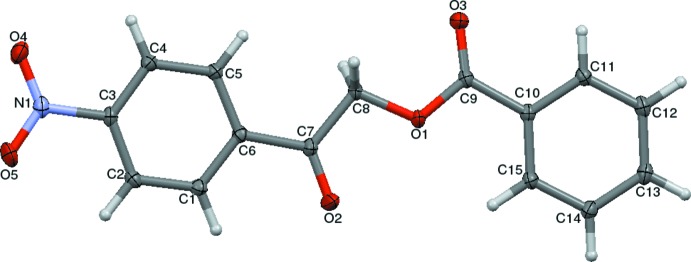
The mol­ecular structure of the title com­pound, with the atom labelling. Displacement ellipsoids are drawn at the 50% probability level.

The mol­ecular structure of the title com­pound is shown in Fig. 1 ▸. The com­pound is com­posed of two aromatic rings linked by a C—C(=O)—O—C(=O) bridge. The unique mol­ecular conformation of this com­pound is characterized by three torsion angles, *viz.* τ_1_ (C11—C10—C9—O3), τ_2_ (C7—C8—O1—C9) and τ_3_ (O2—C7—C8—O1), whereby the τ_1_ value of 9.60 (16)° signifies the apparent coplanarity between the mean planes of the phenyl ring and the adjacent carbonyl groups of the connecting bridge. The τ_2_ value of 174.08 (9)° between the two carbonyl groups indicates an *anti­periplanar* conformation. Likewise, owing to a substitution on the functional group, the title com­pound experiences steric repulsion between the substituent and adjacent carbonyl groups, influencing the torsion angle [τ_3_ = 1.88 (15)°], and it adopts a +*synperiplanar* conformation. The bond lengths and angles are normal and the mol­ecular conformation is characterized by a dihedral angle of 3.09 (5)° between the mean planes of the two aromatic rings indicating that they are coplanar. The nitro group lies almost in the plane of the phenyl ring, as indicated by the torsion angle values of 7.80 (15) and 8.46 (15)° for C4—C3—N1—O4 and C2—C3—N1—O5, respectively.

## Supra­molecular features   

**Figure 2:**
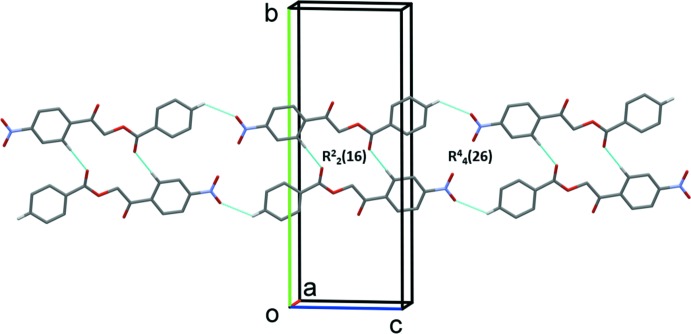
A partial view of the crystal packing of the title com­pound. The hydrogen bonds are shown as dashed lines (Table 1 ▸) and only H atoms H5 and H13 have been included.

**Figure 3:**
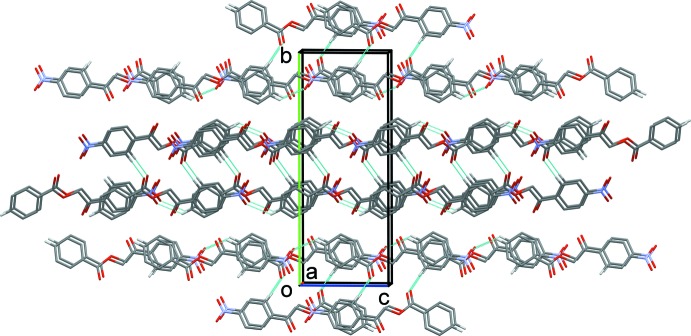
The crystal packing of the title com­pound, viewed along the *c* axis. The hydrogen bonds are shown as dashed lines (Table 1 ▸) and only H atoms H5 and H13 have been included.

In the crystal, there are no classical hydrogen bonds present. However, the structure is stabilized by weak inter­molecular C—H⋯O inter­actions. Specifically, a pair of inter­molecular C5—H5⋯O3^i^ inter­actions stabilize the supra­molecular architecture by forming inversion dimers with an 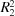
(16) ring motif (Table 1 ▸ and Fig. 2 ▸). The dimers are linked by a further pair of C—H⋯O hydrogen bonds, forming ribbons that enclose 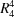
(26) ring motifs (Table 1 ▸ and Fig. 2 ▸). The ribbons are linked by a series of offset π–π inter­actions (Table 2 ▸), forming layers that stack up the *b*-axis direction (Fig. 3 ▸).

## Hirshfeld surface analysis and two-dimensional fingerprint plots   

**Figure 4:**
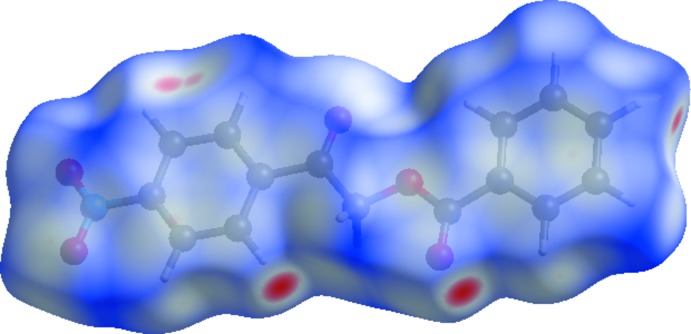
A view of the three-dimensional Hirshfeld surface of the title com­pound mapped over *d*_norm_.

The Hirshfeld surface analysis (Spackman & Jayatilaka, 2009 ▸) and the associated two-dimensional fingerprint plots (McKinnon *et al.*, 2007 ▸) were performed and created with *CrystalExplorer17* (Turner *et al.*, 2017 ▸). Hirshfeld surface analysis enables the visualization of inter­molecular inter­actions by different colours and colour intensity, representing short or long contacts and indicating the relative strengths of the inter­actions. Figs. 4 ▸ and 5 ▸ show the Hirshfeld surfaces mapped over *d*_norm_ (−0.195 to 1.091 a.u.) and shape-index (−1.0 to 1.0 a.u.), respectively.

In Fig. 4 ▸, the dark spots near the C and O atoms result from C—H⋯O inter­actions, which play a significant role in the mol­ecular packing. The Hirshfeld surfaces illustrated in Fig. 4 ▸ also reflect the involvement of different atoms in the inter­molecular inter­actions through the appearance of blue and red regions around the participating atoms, which correspond to positive and negative electrostatic potential, respectively. The shape-index surface clearly shows that the two sides of the mol­ecules are involved in the same contacts with neighbouring mol­ecules and the curvedness plots show flat surface patches characteristic of planar stacking.

**Figure 6:**
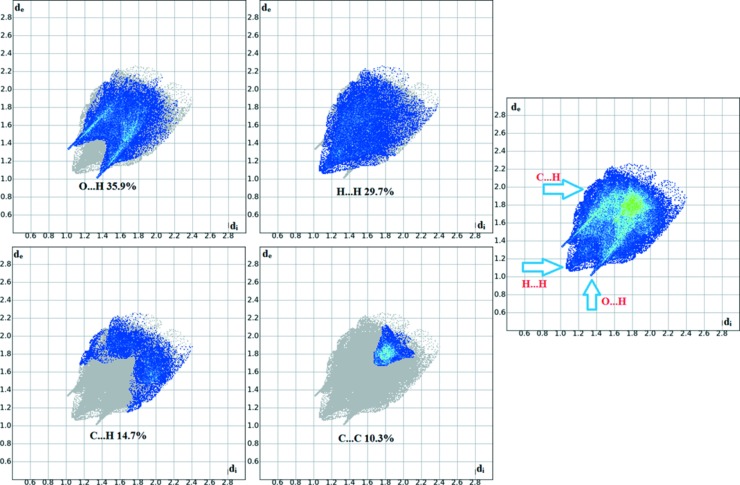
Two-dimensional fingerprint plots of the title com­pound, showing the percentage contributions of all contacts and of individual atom-atom contacts.

The overall two-dimensional fingerprint plot for the title com­pound and those delineated into O—H/H—O, H—H, C—H/H—C and C—C contacts are illustrated in Fig. 6 ▸. The percentage contributions from the different inter­atomic contacts to the Hirshfeld surfaces are O⋯H = 35.9%, H⋯H = 29.7%, C⋯H = 14.7% and C⋯C = 10.3%, and are shown in the two-dimensional fingerprint plots in Fig. 6 ▸. The percentage contributions of other inter­molecular contacts are less than 5% in the Hirshfeld surface mapping.

## Database survey   

A search of the Cambridge Structural Database (CSD, Version 5.40, last update May 2019; Groom *et al.*, 2016 ▸) using 2-oxo-2-phenyl­ethyl benzoate as the main skeleton revealed the presence of 62 structures with different substituents on the terminal phenyl rings (see supplementary information file S1). In these structures, the two aromatic rings are inclined to each other by dihedral angles varying from *ca* 0 to 90°. There are seven structures with a nitro substituent on one of the aromatic rings (see supplementary information file S2). However, there is only one com­pound with the same skeleton as the title com­pound, *i.e.* 2-(biphenyl-4-yl)-2-oxoethyl 4-nitro­benzoate (CSD refcode CISSAB; Kwong *et al.*, 2017 ▸). Here, the two aromatic rings are inclined to each other by *ca* 70.96°, com­pared to an inclination of only 3.09 (5)° in the title com­pound.

## Synthesis and crystallization   

The title com­pound was synthesized as per the procedure of Kumar *et al.* (2014 ▸). A mixture of 2-bromo-1-(4-nitro­phen­yl)ethanone (0.2 g, 0.5 mmol), potassium carbonate (0.087 g, 0.63 mmol) and benzoic acid (0.079 g, 0.65 mmol) in di­methyl­formamide (5 ml) was stirred at room temperature for 2 h. After com­pletion of the reaction, the reaction mixture was poured into ice-cold water. The solid product obtained was filtered off, washed with water and recrystallized from ethanol to give colourless needle-like crystals (m.p. 386–390 K).

## Refinement   

Crystal data, data collection and structure refinement details are summarized in Table 3 ▸. H atoms on C atoms were positioned geometrically (C—H = 0.95–0.99 Å) and refined using a riding model, with *U*_iso_(H) = 1.2*U*_eq_(C).

## Supplementary Material

Crystal structure: contains datablock(s) global, I. DOI: 10.1107/S2056989019013975/su5520sup1.cif

Structure factors: contains datablock(s) I. DOI: 10.1107/S2056989019013975/su5520Isup2.hkl

Supporting information file. DOI: 10.1107/S2056989019013975/su5520Isup5.cml

CSD search file S1. DOI: 10.1107/S2056989019013975/su5520sup3.pdf

CSD search file S2. DOI: 10.1107/S2056989019013975/su5520sup4.pdf

CCDC references: 1449646, 1449646

Additional supporting information:  crystallographic information; 3D view; checkCIF report

## Figures and Tables

**Figure 5:**
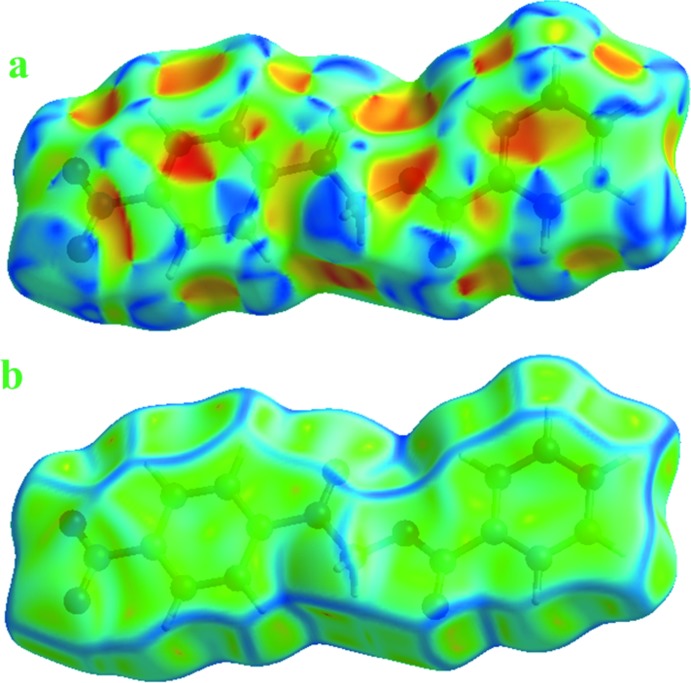
Hirshfeld surface of the title com­pound, mapped over (*a*) the shape-index and (*b*) the curvedness.

**Table 1:** Hydrogen-bond geometry (Å, °)

*D*—H⋯*A*	*D*—H	H⋯*A*	*D*⋯*A*	*D*—H⋯*A*
C5—H5⋯O3^i^	0.95	2.47	3.3967 (14)	164
C13—H13⋯O5^ii^	0.95	2.54	3.2361 (14)	130

Symmetry codes: (i) 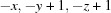; (ii) 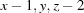.

**Table 2:** π–π contacts (Å, °) in the crystal of the title com­pound *Cg*1 and *Cg*2 are the centroids of rings C1–C6 and C10–C15, respectively.

*Cg*(*I*)	*Cg*(*J*)	*Cg*(*I*)⋯*Cg*(*J*) (Å)	α (°)	β (°)	γ (°)	*CgI*_Perp (Å)	*CgJ*_Perp (Å)	offset (Å)
*Cg*1	*Cg*2^iii^	3.6754 (6)	3.09 (5)	22.5	21.5	3.4199 (4)	3.3948 (4)	1.408
*Cg*1	*Cg*2^iv^	3.7519 (6)	3.09 (5)	27.9	25.1	3.3975 (4)	3.3171 (4)	1.753
*Cg*2	*Cg*1^v^	3.7519 (6)	3.09 (5)	25.1	27.9	3.3171 (4)	3.3975 (4)	1.592
*Cg*2	*Cg*1^vi^	3.6754 (6)	3.09 (5)	21.5	22.5	3.3948 (4)	3.4200 (4)	1.346

Symmetry codes: (iii) *x*, *y*, *z* + 1; (iv) *x* + 1, *y*, *z* + 1; (v) *x* − 1, *y*, *z* − 1; (vi) *x*, *y*, *z* − 1.

**Table 3:** Experimental details

Crystal data	
Chemical formula	C_15_H_11_NO_5_
*M*_r_	285.25
Crystal system, space group	Monoclinic, *P*2_1_/*c*
Temperature (K)	100
*a*, *b*, *c* (Å)	7.3371 (4), 21.0051 (11), 8.3069 (4)
β (°)	102.711 (1)
*V* (Å^3^)	1248.86 (11)
*Z*	4
Radiation type	Mo *K*α
μ (mm^−1^)	0.12
Crystal size (mm)	0.37 × 0.19 × 0.11

Data collection	
Diffractometer	Bruker APEXII DUO CCD area-detector
Absorption correction	Multi-scan (*SADABS*; Bruker, 2012 ▸)
*T*_min_, *T*_max_	0.959, 0.988
No. of measured, independent and observed [*I* > 2σ(*I*)] reflections	14140, 3496, 3133
*R*_int_	0.029
(sin θ/λ)_max_ (Å^−1^)	0.708

Refinement	
*R*[*F* ^2^ > 2σ(*F* ^2^)], *wR*(*F* ^2^), *S*	0.041, 0.120, 1.06
No. of reflections	3696
No. of parameters	190
H-atom treatment	H-atom parameters constrained
Δρ_max_, Δρ_min_ (e Å^−3^)	0.39, −0.30

Computer programs: *APEX2* (Bruker, 2012 ▸), *SAINT* (Bruker, 2012 ▸), *SHELXS97* (Sheldrick, 2008 ▸), *Mercury* (Macrae *et al.*, 2008 ▸), *SHELXL97* (Sheldrick, 2008 ▸), *PLATON* (Spek, 2009 ▸) and *publCIF* (Westrip, 2010 ▸).

## supplementary crystallographic information

### Crystal data

**Table:**

C_15_H_11_NO_5_	*F*(000) = 592
*M**_r_* = 285.25	*D*_x_ = 1.517 Mg m^−^^3^
Monoclinic, *P*2_1_/*c*	Mo *K*α radiation, λ = 0.71073 Å
Hall symbol: -P 2ybc	Cell parameters from 3133 reflections
*a* = 7.3371 (4) Å	θ = 1.9–30.2°
*b* = 21.0051 (11) Å	µ = 0.12 mm^−^^1^
*c* = 8.3069 (4) Å	*T* = 100 K
β = 102.711 (1)°	Needle, colourless
*V* = 1248.86 (11) Å^3^	0.37 × 0.19 × 0.11 mm
*Z* = 4	

### Data collection

**Table:**

Bruker APEXII DUO CCD area-detector diffractometer	3496 independent reflections
Radiation source: Rotating Anode	3133 reflections with *I* > 2σ(*I*)
Graphite monochromator	*R*_int_ = 0.029
Detector resolution: 18.4 pixels mm^-1^	θ_max_ = 30.2°, θ_min_ = 1.9°
φ and ω scans	*h* = −10→10
Absorption correction: multi-scan (SADABS; Bruker, 2012)	*k* = −29→29
*T*_min_ = 0.959, *T*_max_ = 0.988	*l* = −11→11
14140 measured reflections	

### Refinement

**Table:**

Refinement on *F*^2^	Primary atom site location: structure-invariant direct methods
Least-squares matrix: full	Secondary atom site location: difference Fourier map
*R*[*F*^2^ > 2σ(*F*^2^)] = 0.041	Hydrogen site location: inferred from neighbouring sites
*wR*(*F*^2^) = 0.120	H-atom parameters constrained
*S* = 1.06	*w* = 1/[σ^2^(*F*_o_^2^) + (0.0687*P*)^2^ + 0.3119*P*] where *P* = (*F*_o_^2^ + 2*F*_c_^2^)/3
3696 reflections	(Δ/σ)_max_ < 0.001
190 parameters	Δρ_max_ = 0.39 e Å^−^^3^
0 restraints	Δρ_min_ = −0.30 e Å^−^^3^

### Special details

**Table:**

Geometry. Bond distances, angles etc. have been calculated using the rounded fractional coordinates. All su's are estimated from the variances of the (full) variance-covariance matrix. The cell esds are taken into account in the estimation of distances, angles and torsion angles
Refinement. Refinement on F^2^ for ALL reflections except those flagged by the user for potential systematic errors. Weighted R-factors wR and all goodnesses of fit S are based on F^2^, conventional R-factors R are based on F, with F set to zero for negative F^2^. The observed criterion of F^2^ > 2sigma(F^2^) is used only for calculating -R-factor-obs etc. and is not relevant to the choice of reflections for refinement. R-factors based on F^2^ are statistically about twice as large as those based on F, and R-factors based on ALL data will be even larger.

### Fractional atomic coordinates and isotropic or equivalent isotropic displacement parameters (Å^2^)

**Table:**

	*x*	*y*	*z*	*U*_iso_*/*U*_eq_	
O1	0.14827 (11)	0.38115 (4)	0.37625 (9)	0.0160 (2)	
O2	0.34743 (16)	0.30646 (4)	0.58368 (11)	0.0357 (3)	
O3	−0.02999 (13)	0.46698 (4)	0.29186 (10)	0.0227 (3)	
O4	0.55675 (12)	0.44140 (4)	1.40112 (10)	0.0228 (2)	
O5	0.70482 (14)	0.35199 (5)	1.40652 (11)	0.0292 (3)	
N1	0.59732 (13)	0.39281 (5)	1.33492 (11)	0.0167 (2)	
C1	0.47223 (14)	0.31711 (5)	0.92088 (13)	0.0142 (3)	
C2	0.54458 (14)	0.32506 (5)	1.08833 (13)	0.0142 (3)	
C3	0.51595 (14)	0.38305 (5)	1.15843 (12)	0.0129 (2)	
C4	0.41568 (14)	0.43237 (5)	1.06970 (13)	0.0137 (2)	
C5	0.34479 (14)	0.42390 (5)	0.90160 (12)	0.0128 (2)	
C6	0.37472 (13)	0.36653 (5)	0.82655 (12)	0.0123 (2)	
C7	0.30848 (15)	0.35532 (5)	0.64525 (13)	0.0152 (3)	
C8	0.19380 (15)	0.40611 (5)	0.54070 (12)	0.0144 (3)	
C9	0.03139 (14)	0.41587 (5)	0.26299 (12)	0.0132 (2)	
C10	−0.01390 (13)	0.38341 (5)	0.09985 (12)	0.0122 (2)	
C11	−0.11222 (14)	0.41756 (5)	−0.03566 (13)	0.0140 (3)	
C12	−0.16055 (14)	0.38880 (5)	−0.18959 (13)	0.0159 (3)	
C13	−0.11199 (15)	0.32571 (5)	−0.20797 (13)	0.0168 (3)	
C14	−0.01248 (15)	0.29164 (5)	−0.07323 (13)	0.0162 (3)	
C15	0.03811 (14)	0.32043 (5)	0.08058 (13)	0.0138 (3)	
H1	0.48900	0.27770	0.86970	0.0170*	
H2	0.61170	0.29180	1.15300	0.0170*	
H4	0.39580	0.47110	1.12250	0.0160*	
H5	0.27610	0.45710	0.83790	0.0150*	
H8A	0.07880	0.41510	0.58050	0.0170*	
H8B	0.26650	0.44600	0.54450	0.0170*	
H11	−0.14620	0.46060	−0.02260	0.0170*	
H12	−0.22660	0.41220	−0.28210	0.0190*	
H13	−0.14680	0.30570	−0.31290	0.0200*	
H14	0.02090	0.24860	−0.08660	0.0190*	
H15	0.10770	0.29740	0.17220	0.0170*	

### Atomic displacement parameters (Å^2^)

**Table:**

	*U*^11^	*U*^22^	*U*^33^	*U*^12^	*U*^13^	*U*^23^
O1	0.0200 (4)	0.0170 (4)	0.0090 (3)	0.0052 (3)	−0.0008 (3)	−0.0008 (3)
O2	0.0581 (7)	0.0226 (4)	0.0179 (4)	0.0205 (4)	−0.0097 (4)	−0.0078 (3)
O3	0.0325 (5)	0.0174 (4)	0.0160 (4)	0.0090 (3)	0.0003 (3)	−0.0014 (3)
O4	0.0256 (4)	0.0266 (4)	0.0150 (4)	0.0018 (3)	0.0018 (3)	−0.0064 (3)
O5	0.0366 (5)	0.0313 (5)	0.0149 (4)	0.0113 (4)	−0.0048 (4)	0.0036 (3)
N1	0.0159 (4)	0.0219 (4)	0.0115 (4)	0.0000 (3)	0.0015 (3)	0.0007 (3)
C1	0.0155 (5)	0.0123 (4)	0.0142 (5)	−0.0001 (3)	0.0017 (4)	0.0004 (3)
C2	0.0142 (5)	0.0138 (4)	0.0137 (5)	0.0008 (3)	0.0012 (4)	0.0032 (3)
C3	0.0120 (4)	0.0171 (4)	0.0093 (4)	−0.0018 (3)	0.0015 (3)	0.0007 (3)
C4	0.0142 (4)	0.0138 (4)	0.0128 (4)	0.0007 (3)	0.0026 (4)	−0.0012 (3)
C5	0.0130 (4)	0.0128 (4)	0.0119 (4)	0.0016 (3)	0.0010 (3)	0.0007 (3)
C6	0.0117 (4)	0.0126 (4)	0.0119 (4)	−0.0012 (3)	0.0012 (3)	−0.0005 (3)
C7	0.0171 (5)	0.0145 (4)	0.0120 (4)	0.0007 (4)	−0.0012 (4)	−0.0007 (3)
C8	0.0184 (5)	0.0149 (4)	0.0089 (4)	0.0024 (4)	0.0010 (4)	−0.0004 (3)
C9	0.0139 (4)	0.0142 (4)	0.0112 (4)	−0.0001 (3)	0.0020 (4)	0.0019 (3)
C10	0.0115 (4)	0.0144 (4)	0.0106 (4)	−0.0010 (3)	0.0021 (3)	0.0002 (3)
C11	0.0136 (4)	0.0151 (4)	0.0129 (5)	0.0012 (3)	0.0022 (4)	0.0021 (3)
C12	0.0142 (5)	0.0207 (5)	0.0118 (5)	−0.0004 (4)	0.0007 (4)	0.0030 (4)
C13	0.0180 (5)	0.0209 (5)	0.0109 (4)	−0.0024 (4)	0.0022 (4)	−0.0011 (4)
C14	0.0203 (5)	0.0156 (5)	0.0128 (5)	0.0002 (4)	0.0041 (4)	−0.0012 (4)
C15	0.0157 (5)	0.0142 (4)	0.0113 (4)	0.0004 (3)	0.0027 (4)	0.0015 (3)

### Geometric parameters (Å, º)

**Table:**

O1—C8	1.4324 (12)	C10—C15	1.3958 (15)
O1—C9	1.3407 (13)	C11—C12	1.3879 (15)
O2—C7	1.2087 (14)	C12—C13	1.3894 (15)
O3—C9	1.2085 (14)	C13—C14	1.3923 (15)
O4—N1	1.2267 (13)	C14—C15	1.3883 (15)
O5—N1	1.2266 (14)	C1—H1	0.9500
N1—C3	1.4706 (13)	C2—H2	0.9500
C1—C2	1.3855 (15)	C4—H4	0.9500
C1—C6	1.3988 (15)	C5—H5	0.9500
C2—C3	1.3859 (15)	C8—H8A	0.9900
C3—C4	1.3853 (15)	C8—H8B	0.9900
C4—C5	1.3901 (14)	C11—H11	0.9500
C5—C6	1.3962 (15)	C12—H12	0.9500
C6—C7	1.4959 (14)	C13—H13	0.9500
C7—C8	1.5096 (15)	C14—H14	0.9500
C9—C10	1.4878 (14)	C15—H15	0.9500
C10—C11	1.3945 (14)		
			
C8—O1—C9	116.69 (8)	C12—C13—C14	120.25 (10)
O4—N1—O5	123.90 (9)	C13—C14—C15	120.18 (10)
O4—N1—C3	118.53 (9)	C10—C15—C14	119.60 (10)
O5—N1—C3	117.56 (9)	C2—C1—H1	120.00
C2—C1—C6	120.58 (10)	C6—C1—H1	120.00
C1—C2—C3	117.89 (10)	C1—C2—H2	121.00
N1—C3—C2	118.40 (9)	C3—C2—H2	121.00
N1—C3—C4	118.54 (9)	C3—C4—H4	121.00
C2—C3—C4	123.06 (9)	C5—C4—H4	121.00
C3—C4—C5	118.44 (10)	C4—C5—H5	120.00
C4—C5—C6	119.88 (9)	C6—C5—H5	120.00
C1—C6—C5	120.11 (9)	O1—C8—H8A	111.00
C1—C6—C7	117.39 (9)	O1—C8—H8B	111.00
C5—C6—C7	122.50 (9)	C7—C8—H8A	111.00
O2—C7—C6	120.33 (10)	C7—C8—H8B	111.00
O2—C7—C8	120.69 (10)	H8A—C8—H8B	109.00
C6—C7—C8	118.98 (9)	C10—C11—H11	120.00
O1—C8—C7	105.87 (8)	C12—C11—H11	120.00
O1—C9—O3	123.56 (9)	C11—C12—H12	120.00
O1—C9—C10	111.72 (9)	C13—C12—H12	120.00
O3—C9—C10	124.72 (9)	C12—C13—H13	120.00
C9—C10—C11	118.15 (9)	C14—C13—H13	120.00
C9—C10—C15	121.80 (9)	C13—C14—H14	120.00
C11—C10—C15	120.06 (9)	C15—C14—H14	120.00
C10—C11—C12	120.11 (10)	C10—C15—H15	120.00
C11—C12—C13	119.78 (10)	C14—C15—H15	120.00
			
C9—O1—C8—C7	174.08 (9)	C1—C6—C7—C8	176.74 (10)
C8—O1—C9—O3	2.69 (15)	C5—C6—C7—O2	175.36 (11)
C8—O1—C9—C10	−176.70 (9)	C5—C6—C7—C8	−4.18 (15)
O4—N1—C3—C2	−172.74 (10)	O2—C7—C8—O1	1.88 (15)
O4—N1—C3—C4	7.80 (15)	C6—C7—C8—O1	−178.58 (9)
O5—N1—C3—C2	8.46 (15)	O1—C9—C10—C11	−171.02 (9)
O5—N1—C3—C4	−171.00 (10)	O1—C9—C10—C15	9.44 (14)
C6—C1—C2—C3	0.43 (16)	O3—C9—C10—C11	9.60 (16)
C2—C1—C6—C5	−1.83 (16)	O3—C9—C10—C15	−169.95 (11)
C2—C1—C6—C7	177.27 (10)	C9—C10—C11—C12	−178.79 (10)
C1—C2—C3—N1	−178.03 (9)	C15—C10—C11—C12	0.77 (15)
C1—C2—C3—C4	1.41 (16)	C9—C10—C15—C14	178.01 (10)
N1—C3—C4—C5	177.65 (9)	C11—C10—C15—C14	−1.52 (15)
C2—C3—C4—C5	−1.79 (16)	C10—C11—C12—C13	0.53 (16)
C3—C4—C5—C6	0.33 (15)	C11—C12—C13—C14	−1.06 (16)
C4—C5—C6—C1	1.43 (15)	C12—C13—C14—C15	0.30 (17)
C4—C5—C6—C7	−177.62 (10)	C13—C14—C15—C10	0.99 (16)
C1—C6—C7—O2	−3.72 (16)		

### Hydrogen-bond geometry (Å, º)

**Table:**

*D*—H···*A*	*D*—H	H···*A*	*D*···*A*	*D*—H···*A*
C5—H5···O3^i^	0.95	2.47	3.3967 (14)	164
C13—H13···O5^ii^	0.95	2.54	3.2361 (14)	130

Symmetry codes: (i) −*x*, −*y*+1, −*z*+1; (ii) *x*−1, *y*, *z*−2.
