# Supplementary material for: Crystal structure and Hirshfeld surface analysis of 2-(4-nitro­phen­yl)-2-oxoethyl benzoate
Source: Acta Crystallogr E Crystallogr Commun. 2019 Oct 22;75(Pt 11):1719–23. doi: 10.1107/S2056989019013975 (PMC6829730; doi:10.1107/S2056989019013975)

Search Overview

Search:	search1
Date/Time done:	Sun Oct 13 16:24:06 2019
Database(s):	CSD version 5.40 updates (Feb 2019)<br>CSD version 5.40 (November 2018)<br>CSD version 5.40 updates (May 2019)<br>CSD version 5.40 updates (Aug 2019)
Restriction Info:	No refcode restrictions applied
Filters:	None
Percentage Completed:	100%
Number of Hits:	62

Single query used. Search found structures that:

match

Query 1

Query 1

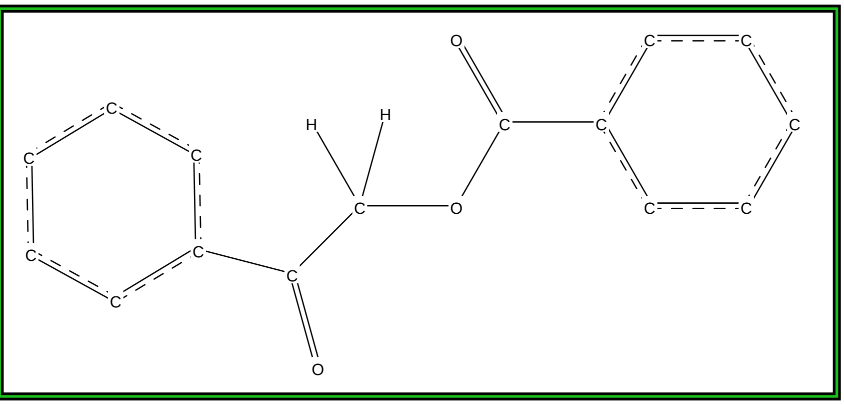

Search: search1 (Sun Oct 13 16:24:06 2019): Hits 1-4

CISRUU

Reference: Huey Chong Kwong, C.S.Chidan Kumar, Siau Hui Mah, Tze Shyang Chia, Ching Kheng Quah, Zi Han Loh, S.Chandraju, Gin Keat Lim (2017) *Plos One* ,**12,**e0170117

Formula: $C_{21}\,H_{15}\,N_1\,O_5$

Compound Name: 2-(biphenyl-4-yl)-2-oxoethyl 3-nitrobenzoate

Space Group:	P-1	***Cell:***	***a***	4.933(0)	***b***	18.312(1)	***c***	19.167(1)
Space Group No.:	2	***(Å,°)***	α	103.22(0)	β	97.41(0)	γ	89.98(0)

R-Factor (%): 4.21 ***Temperature(K):*** 100 ***Density(g/cm^3):*** 1.437

Parameters

Fragment 1
ANG1 (A) 64.060
ANG2 (A) 69.594
ANG3 (A) 65.756

Fragment 2
ANG1 (A) 64.087
ANG2 (A) 69.652
ANG3 (A) 65.704

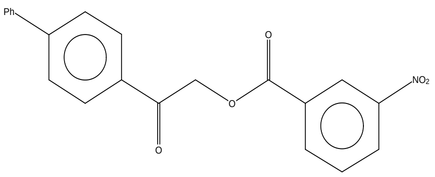

CISSAB

Reference: Huey Chong Kwong, C.S.Chidan Kumar, Siau Hui Mah, Tze Shyang Chia, Ching Kheng Quah, Zi Han Loh, S.Chandraju, Gin Keat Lim (2017) *Plos One* ,**12,**e0170117

Formula: $C_{21}\,H_{15}\,N_1\,O_5$

Compound Name: 2-(biphenyl-4-yl)-2-oxoethyl 4-nitrobenzoate

Space Group:	P21/c	***Cell:***	***a***	8.681(1)	***b***	5.555(0)	***c***	35.401(5)
Space Group No.:	14	***(Å,°)***	α	90.00	β	90.12(0)	γ	90.00

R-Factor (%): 5.26 ***Temperature(K):*** 293 ***Density(g/cm^3):*** 1.406

Parameters

Fragment 1
ANG1 (A) 70.959
ANG2 (A) 42.607
ANG3 (A) 82.524

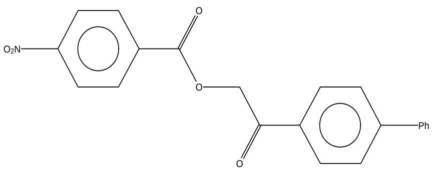

CISSEF

Reference: Huey Chong Kwong, C.S.Chidan Kumar, Siau Hui Mah, Tze Shyang Chia, Ching Kheng Quah, Zi Han Loh, S.Chandraju, Gin Keat Lim (2017) *Plos One* ,**12,**e0170117

Formula: $C_{21}\,H_{17}\,N_1\,O_3$

Compound Name: 2-(biphenyl-4-yl)-2-oxoethyl 2-aminobenzoate

Space Group:	Pbca	***Cell:***	***a***	10.661(1)	***b***	9.824(1)	***c***	32.235(4)
Space Group No.:	61	***(Å,°)***	α	90.00	β	90.00	γ	90.00

R-Factor (%): 4.82 ***Temperature(K):*** 294 ***Density(g/cm^3):*** 1.304

Parameters

Fragment 1
ANG1 (A) 52.331
ANG2 (A) 52.868
ANG3 (A) 55.822

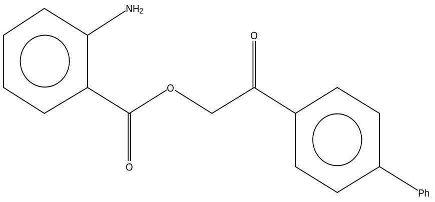

CISSIJ

Reference: Huey Chong Kwong, C.S.Chidan Kumar, Siau Hui Mah, Tze Shyang Chia, Ching Kheng Quah, Zi Han Loh, S.Chandraju, Gin Keat Lim (2017) *Plos One* ,**12,**e0170117

Formula: $C_{21}\,H_{17}\,N_1\,O_3$

Compound Name: 2-(biphenyl-4-yl)-2-oxoethyl 4-aminobenzoate

Space Group:	P21	***Cell:***	***a***	8.330(0)	***b***	5.065(0)	***c***	19.467(1)
Space Group No.:	4	***(Å,°)***	α	90.00	β	95.71(0)	γ	90.00

R-Factor (%): 3.63 ***Temperature(K):*** 100 ***Density(g/cm^3):*** 1.347

Parameters

Fragment 1
ANG1 (A) 78.446
ANG2 (A) 70.856
ANG3 (A) 65.332

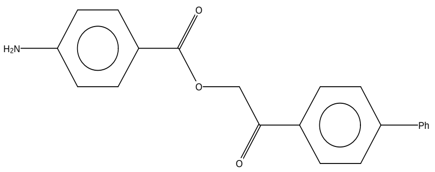

Search: search1 (Sun Oct 13 16:24:06 2019): Hits 5-8

GESBUE01

Reference: Huey Chong Kwong, C.S.Chidan Kumar, Siau Hui Mah, Tze Shyang Chia, Ching Kheng Quah, Zi Han Loh, S.Chandraju, Gin Keat Lim (2017) *Plos One* ,**12,**e0170117

Formula: $C_{21}\,H_{17}\,N_{1}\,O_{3}$

Compound Name: 2-(biphenyl-4-yl)-2-oxoethyl 3-aminobenzoate

Space Group:	P-1	***Cell:***	***a***	5.293(0)	***b***	11.682(1)	***c***	13.404(1)
Space Group No.:	2	***(Å,°)***	α	87.86(0)	β	83.98(0)	γ	87.70(0)

R-Factor (%): 5.45 ***Temperature(K):*** 294 ***Density(g/cm³):*** 1.337

Parameters

Fragment 1

**ANG1 (A)**	75.690
**ANG2 (A)**	45.492
**ANG3 (A)**	61.521

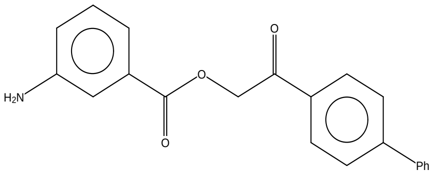

JIVHAA

Reference: Huey Chong Kwong, C.S.Chidan Kumar, Siau Hui Mah, Tze Shyang Chia, Ching Kheng Quah, Zi Han Loh, S.Chandraju, Gin Keat Lim (2017) *Plos One* ,**12,**e0170117

Formula: $C_{21}\,H_{15}\,Cl_{1}\,O_{3}$

Compound Name: 2-(biphenyl-4-yl)-2-oxoethyl 2-chlorobenzoate

Space Group:	Pbca	***Cell:***	***a***	11.397(1)	***b***	8.036(1)	***c***	36.509(6)
Space Group No.:	61	***(Å,°)***	α	90.00	β	90.00	γ	90.00

R-Factor (%): 5.50 ***Temperature(K):*** 100 ***Density(g/cm³):*** 1.394

Parameters

Fragment 1

**ANG1 (A)**	74.357
**ANG2 (A)**	86.176
**ANG3 (A)**	52.373

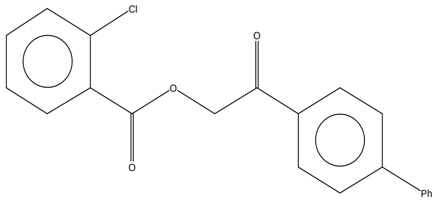

JIVHEE

Reference: Huey Chong Kwong, C.S.Chidan Kumar, Siau Hui Mah, Tze Shyang Chia, Ching Kheng Quah, Zi Han Loh, S.Chandraju, Gin Keat Lim (2017) *Plos One* ,**12,**e0170117

Formula: $C_{22}\,H_{18}\,O_{3}$

Compound Name: 2-(biphenyl-4-yl)-2-oxoethyl 3-methylbenzoate

Space Group:	P21	***Cell:***	***a***	5.288(1)	***b***	8.975(2)	***c***	17.996(4)
Space Group No.:	4	***(Å,°)***	α	90.00	β	93.83(0)	γ	90.00

R-Factor (%): 4.60 ***Temperature(K):*** 294 ***Density(g/cm³):*** 1.288

Parameters

Fragment 1

**ANG1 (A)**	77.591
**ANG2 (A)**	61.341
**ANG3 (A)**	54.645

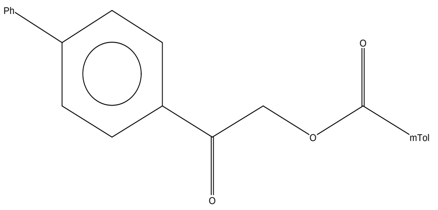

JIVHII

Reference: Huey Chong Kwong, C.S.Chidan Kumar, Siau Hui Mah, Tze Shyang Chia, Ching Kheng Quah, Zi Han Loh, S.Chandraju, Gin Keat Lim (2017) *Plos One* ,**12,**e0170117

Formula: $C_{21}\,H_{15}\,Cl_{1}\,O_{3}$

Compound Name: 2-(biphenyl-4-yl)-2-oxoethyl 3-chlorobenzoate

Space Group:	P21/c	***Cell:***	***a***	5.026(0)	***b***	9.277(1)	***c***	36.530(4)
Space Group No.:	14	***(Å,°)***	α	90.00	β	91.23(0)	γ	90.00

R-Factor (%): 4.76 ***Temperature(K):*** 294 ***Density(g/cm³):*** 1.368

Parameters

Fragment 1

**ANG1 (A)**	56.619
**ANG2 (A)**	81.410
**ANG3 (A)**	64.521

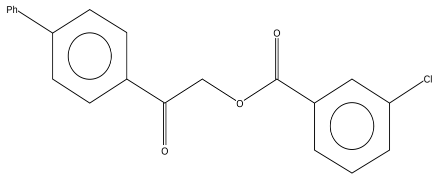

Search: search1 (Sun Oct 13 16:24:06 2019): Hits 9-12

JIVHOO

Reference: Huey Chong Kwong, C.S.Chidan Kumar, Siau Hui Mah, Tze Shyang Chia, Ching Kheng Quah, Zi Han Loh, S.Chandraju, Gin Keat Lim (2017) *Plos One* ,**12,**e0170117

Formula: $C_{21} H_{14} Cl_2 O_3$

Compound Name: 2-(biphenyl-4-yl)-2-oxoethyl 2,4-dichlorobenzoate

Space Group:	P21/c	***Cell:***	***a***	12.543(1)	***b***	8.658(0)	***c***	17.446(1)
Space Group No.:	14	***(Å,°)***	α	90.00	β	108.99(0)	γ	90.00

R-Factor (%): 5.06 ***Temperature(K):*** 294 ***Density(g/cm^3):*** 1.428

Parameters

Fragment 1

ANG1 (A) 77.636
ANG2 (A) 53.831
ANG3 (A) 65.242

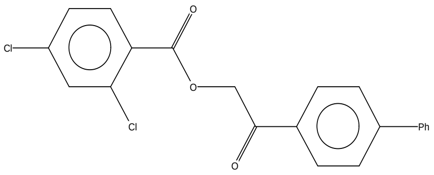

JIVHUU

Reference: Huey Chong Kwong, C.S.Chidan Kumar, Siau Hui Mah, Tze Shyang Chia, Ching Kheng Quah, Zi Han Loh, S.Chandraju, Gin Keat Lim (2017) *Plos One* ,**12,**e0170117

Formula: $C_{22} H_{18} O_4$

Compound Name: 2-(biphenyl-4-yl)-2-oxoethyl 3-methoxybenzoate

Space Group:	P21/c	***Cell:***	***a***	10.725(0)	***b***	9.072(0)	***c***	17.368(1)
Space Group No.:	14	***(Å,°)***	α	90.00	β	93.34(0)	γ	90.00

R-Factor (%): 4.23 ***Temperature(K):*** 100 ***Density(g/cm^3):*** 1.364

Parameters

Fragment 1

ANG1 (A) 76.009
ANG2 (A) 56.765
ANG3 (A) 68.811

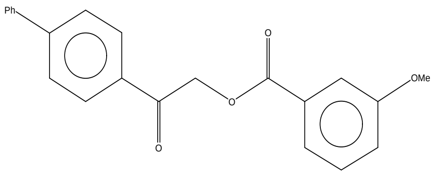

JIVJAC

Reference: Huey Chong Kwong, C.S.Chidan Kumar, Siau Hui Mah, Tze Shyang Chia, Ching Kheng Quah, Zi Han Loh, S.Chandraju, Gin Keat Lim (2017) *Plos One* ,**12,**e0170117

Formula: $C_{21} H_{15} Cl_1 O_3$

Compound Name: 2-(biphenyl-4-yl)-2-oxoethyl 4-chlorobenzoate

Space Group:	Pbca	***Cell:***	***a***	9.715(0)	***b***	9.439(0)	***c***	36.285(2)
Space Group No.:	61	***(Å,°)***	α	90.00	β	90.00	γ	90.00

R-Factor (%): 3.91 ***Temperature(K):*** 100 ***Density(g/cm^3):*** 1.401

Parameters

Fragment 1

ANG1 (A) 43.718
ANG2 (A) 56.942
ANG3 (A) 51.884

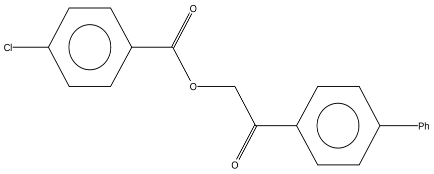

JIVJEG

Reference: Huey Chong Kwong, C.S.Chidan Kumar, Siau Hui Mah, Tze Shyang Chia, Ching Kheng Quah, Zi Han Loh, S.Chandraju, Gin Keat Lim (2017) *Plos One* ,**12,**e0170117

Formula: $C_{22} H_{18} O_4$

Compound Name: 2-(biphenyl-4-yl)-2-oxoethyl 2-methoxybenzoate

Space Group:	Pbca	***Cell:***	***a***	17.341(2)	***b***	8.920(1)	***c***	22.412(3)
Space Group No.:	61	***(Å,°)***	α	90.00	β	90.00	γ	90.00

R-Factor (%): 5.16 ***Temperature(K):*** 100 ***Density(g/cm^3):*** 1.327

Parameters

Fragment 1

ANG1 (A) 62.833
ANG2 (A) 55.977
ANG3 (A) 80.097

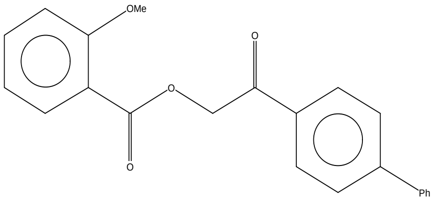

Search: search1 (Sun Oct 13 16:24:06 2019): Hits 13-16

JIVJIK

Reference: Huey Chong Kwong, C.S.Chidan Kumar, Siau Hui Mah, Tze Shyang Chia, Ching Kheng Quah, Zi Han Loh, S.Chandraju, Gin Keat Lim (2017) *Plos One* ,**12,**e0170117

Formula: $C_{22} H_{18} O_4$

Compound Name: 2-(biphenyl-4-yl)-2-oxoethyl 4-methoxybenzoate

Space Group:	P21	***Cell:***	***a***	8.659(1)	***b***	5.269(0)	***c***	19.270(3)
Space Group No.:	4	***(Å,°)***	α	90.00	β	91.78(0)	γ	90.00

R-Factor (%): 4.43 ***Temperature(K):*** 294 ***Density(g/cm³):*** 1.309

Parameters

Fragment 1

**ANG1 (A)**	75.291
**ANG2 (A)**	58.878
**ANG3 (A)**	54.645

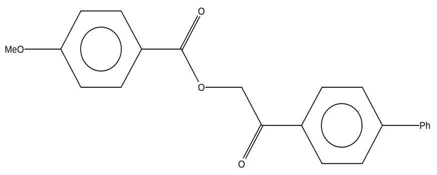

JIVJOQ

Reference: Huey Chong Kwong, C.S.Chidan Kumar, Siau Hui Mah, Tze Shyang Chia, Ching Kheng Quah, Zi Han Loh, S.Chandraju, Gin Keat Lim (2017) *Plos One* ,**12,**e0170117

Formula: $C_{21} H_{15} N_1 O_5$

Compound Name: 2-(biphenyl-4-yl)-2-oxoethyl 2-nitrobenzoate

Space Group:	Pna21	***Cell:***	***a***	9.844(1)	***b***	32.312(5)	***c***	5.368(0)
Space Group No.:	33	***(Å,°)***	α	90.00	β	90.00	γ	90.00

R-Factor (%): 5.54 ***Temperature(K):*** 100 ***Density(g/cm³):*** 1.406

Parameters

Fragment 1

**ANG1 (A)**	11.771
**ANG2 (A)**	58.652
**ANG3 (A)**	61.685

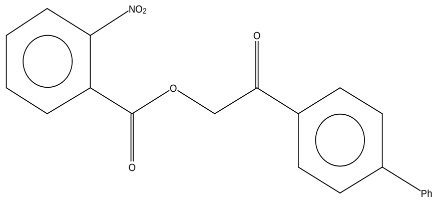

AZULUD

Reference: H.-K.Fun, T.Shahani, B.Garudachari, A.M.Isloor, K.N.Shivananda (2011) *Acta Crystallogr.,Sect.E:Struct.Rep.Online* ,**67,** o2682

Formula: $C_{15} H_{11} Cl_1 O_4$

Compound Name: 2-(4-Chlorophenyl)-2-oxoethyl 4-hydroxybenzoate

Space Group:	P21	***Cell:***	***a***	5.531(1)	***b***	8.132(1)	***c***	14.857(2)
Space Group No.:	4	***(Å,°)***	α	90.00	β	95.12(0)	γ	90.00

R-Factor (%): 4.43 ***Temperature(K):*** 296 ***Density(g/cm³):*** 1.451

Parameters

Fragment 1

**ANG1 (A)**	65.702
**ANG2 (A)**	51.592
**ANG3 (A)**	53.658

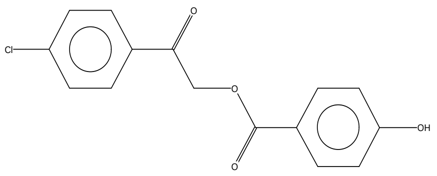

BOQXOW

Reference: I.Khan, A.Ibrar, S.Hameed, J.M.White, J.Simpson (2014) *Acta Crystallogr.,Sect.E:Struct.Rep.Online* ,**70,**301

Formula: $C_{15} H_{10} Br_1 Cl_1 O_3$

Compound Name: 2-(4-chlorophenyl)-2-oxoethyl 3-bromobenzoate

Space Group:	P-1	***Cell:***	***a***	6.680(0)	***b***	10.024(0)	***c***	10.785(0)
Space Group No.:	2	***(Å,°)***	α	90.98(0)	β	107.57(0)	γ	92.14(0)

R-Factor (%): 2.66 ***Temperature(K):*** 130 ***Density(g/cm³):*** 1.708

Parameters

Fragment 1

**ANG1 (A)**	86.110
**ANG2 (A)**	50.650
**ANG3 (A)**	79.121

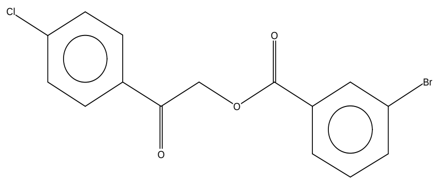

Search: search1 (Sun Oct 13 16:24:06 2019): Hits 17-20

CIQNEW

Reference: Tao Ji, Xi-Lin Wang, Yu-Xing Gao, Guo Tang, Yu-Fen Zhao (2007) *Acta Crystallogr.,Sect.E:Struct.Rep.Online* ,**63,** o4766

Formula: $C_{16} H_{14} O_4$

Compound Name: Benzoylmethyl 4-methoxybenzoate

Space Group: P21/n ***Cell:*** ***a*** 8.062(0) ***b*** 9.667(0) ***c*** 17.182(0)
Space Group No.: 14 ***(Å,°)*** α 90.00 β 91.53(0) γ 90.00

R-Factor (%): 3.46 ***Temperature(K):*** 298 ***Density(g/cm³):*** 1.341

Parameters

Fragment 1
ANG1 (A) 71.209
ANG2 (A) 59.553
ANG3 (A) 53.755

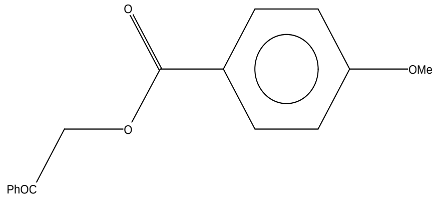

CIXVUC

Reference: C.S.C.Kumar, Tze Shyang Chia, S.Chandraju, Chin Wei Ooi, Ching Kheng Quah, Hoong-Kun Fun (2014) *Z.Krist.Cryst.Mater.* ,**229,**328

Formula: $C_{15} H_{10} Br_1 Cl_1 O_3$

Compound Name: 2-(4-Bromophenyl)-2-oxoethyl 3-chlorobenzoate

Space Group: P-1 ***Cell:*** ***a*** 6.722(0) ***b*** 10.194(1) ***c*** 10.889(1)
Space Group No.: 2 ***(Å,°)*** α 91.67(0) β 107.64(0) γ 90.91(0)

R-Factor (%): 2.64 ***Temperature(K):*** 296 ***Density(g/cm³):*** 1.653

Parameters

Fragment 1
ANG1 (A) 85.170
ANG2 (A) 51.606
ANG3 (A) 77.905

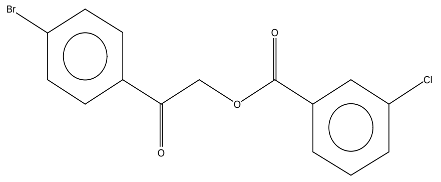

CIXWAJ

Reference: C.S.C.Kumar, Tze Shyang Chia, S.Chandraju, Chin Wei Ooi, Ching Kheng Quah, Hoong-Kun Fun (2014) *Z.Krist.Cryst.Mater.* ,**229,**328

Formula: $C_{15} H_9 Br_1 Cl_2 O_3$

Compound Name: 2-(4-Bromophenyl)-2-oxoethyl 2,4-dichlorobenzoate

Space Group: C2/c ***Cell:*** ***a*** 28.309(2) ***b*** 12.132(0) ***c*** 8.798(0)
Space Group No.: 15 ***(Å,°)*** α 90.00 β 97.71(0) γ 90.00

R-Factor (%): 4.45 ***Temperature(K):*** 296 ***Density(g/cm³):*** 1.721

Parameters

Fragment 1
ANG1 (A) 6.166
ANG2 (A) 6.156
ANG3 (A) 0.062

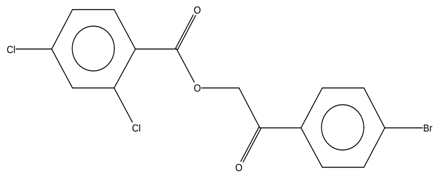

CIXWEN

Reference: C.S.C.Kumar, Tze Shyang Chia, S.Chandraju, Chin Wei Ooi, Ching Kheng Quah, Hoong-Kun Fun (2014) *Z.Krist.Cryst.Mater.* ,**229,**328

Formula: $C_{15} H_{12} Br_1 N_1 O_3$

Compound Name: 2-(4-Bromophenyl)-2-oxoethyl 2-aminobenzoate

Space Group: P21/c ***Cell:*** ***a*** 15.295(2) ***b*** 9.870(1) ***c*** 9.413(1)
Space Group No.: 14 ***(Å,°)*** α 90.00 β 93.62(0) γ 90.00

R-Factor (%): 4.87 ***Temperature(K):*** 296 ***Density(g/cm³):*** 1.565

Parameters

Fragment 1
ANG1 (A) 83.028
ANG2 (A) 70.499
ANG3 (A) 61.438

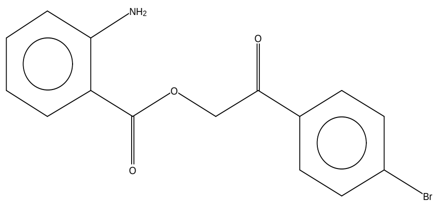

Search: search1 (Sun Oct 13 16:24:06 2019): Hits 21-24

CIXWIR

Reference: C.S.C.Kumar, Tze Shyang Chia, S.Chandraju, Chin Wei Ooi, Ching Kheng Quah, Hoong-Kun Fun (2014) *Z.Krist.Cryst.Mater.* ,**229,**328

Formula: $C_{15} H_{12} Br_1 N_1 O_3$

Compound Name: 2-(4-Bromophenyl)-2-oxoethyl 3-aminobenzoate

Space Group: P21/c ***Cell:*** ***a*** 8.215(1) ***b*** 10.592(1) ***c*** 17.117(1)
Space Group No.: 14 ***(Å,°)*** α 90.00 β 110.95(0) γ 90.00

R-Factor (%): 4.78 ***Temperature(K):*** 296 ***Density(g/cm³):*** 1.596

Parameters

Fragment 1
ANG1 (A) 84.091
ANG2 (A) 49.757
ANG3 (A) 73.834

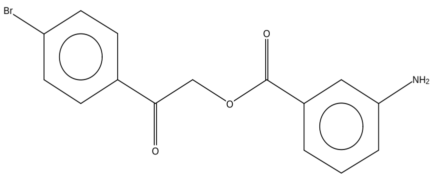

CIYCAQ

Reference: C.S.C.Kumar, Tze Shyang Chia, S.Chandraju, Chin Wei Ooi, Ching Kheng Quah, Hoong-Kun Fun (2014) *Z.Krist.Cryst.Mater.* ,**229,**328

Formula: $C_{15} H_{10} Br_1 N_1 O_5$

Compound Name: 2-(4-Bromophenyl)-2-oxoethyl 2-nitrobenzoate

Space Group: P21/c ***Cell:*** ***a*** 15.596(1) ***b*** 25.103(3) ***c*** 7.254(0)
Space Group No.: 14 ***(Å,°)*** α 90.00 β 99.44(0) γ 90.00

R-Factor (%): 3.83 ***Temperature(K):*** 100 ***Density(g/cm³):*** 1.727

Parameters

Fragment 1
ANG1 (A) 24.998
ANG2 (A) 57.508
ANG3 (A) 65.709

Fragment 2
ANG1 (A) 69.517
ANG2 (A) 40.068
ANG3 (A) 89.505

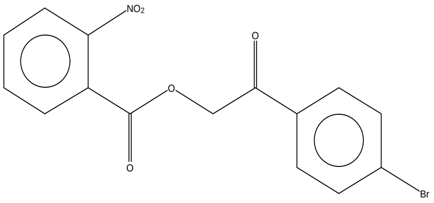

CIYCEU

Reference: C.S.C.Kumar, Tze Shyang Chia, S.Chandraju, Chin Wei Ooi, Ching Kheng Quah, Hoong-Kun Fun (2014) *Z.Krist.Cryst.Mater.* ,**229,**328

Formula: $C_{15} H_{10} Br_1 N_1 O_5$

Compound Name: 2-(4-Bromophenyl)-2-oxoethyl 3-nitrobenzoate

Space Group: P-1 ***Cell:*** ***a*** 9.311(0) ***b*** 12.098(1) ***c*** 14.175(1)
Space Group No.: 2 ***(Å,°)*** α 100.61(0) β 100.35(0) γ 105.99(0)

R-Factor (%): 5.48 ***Temperature(K):*** 296 ***Density(g/cm³):*** 1.653

Parameters

Fragment 1
ANG1 (A) 4.742
ANG2 (A) 1.620
ANG3 (A) 4.446

Fragment 2
ANG1 (A) 7.368
ANG2 (A) 3.781
ANG3 (A) 4.080

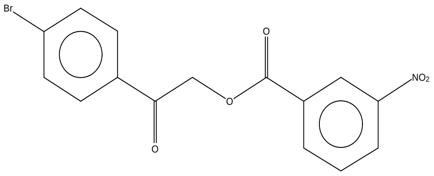

CIYCIY

Reference: C.S.C.Kumar, Tze Shyang Chia, S.Chandraju, Chin Wei Ooi, Ching Kheng Quah, Hoong-Kun Fun (2014) *Z.Krist.Cryst.Mater.* ,**229,**328

Formula: $C_{15} H_{10} Br_1 N_1 O_5$

Compound Name: 2-(4-Bromophenyl)-2-oxoethyl 4-nitrobenzoate

Space Group: P21/c ***Cell:*** ***a*** 14.896(0) ***b*** 12.707(0) ***c*** 7.371(0)
Space Group No.: 14 ***(Å,°)*** α 90.00 β 96.20(0) γ 90.00

R-Factor (%): 4.45 ***Temperature(K):*** 100 ***Density(g/cm³):*** 1.744

Parameters

Fragment 1
ANG1 (A) 82.031
ANG2 (A) 38.088
ANG3 (A) 87.661

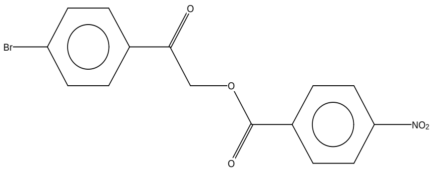

Search: search1 (Sun Oct 13 16:24:06 2019): Hits 25-28

CIYCOE

Reference:	C.S.C.Kumar, Tze Shyang Chia, S.Chandraju, Chin Wei Ooi, Ching Kheng Quah, Hoong-Kun Fun (2014) *Z.Krist.Cryst.Mater.* ,**229,**328
Formula:	C_{15} H_{12} Br_1 N_1 O_3
Compound Name:	2-(4-Bromophenyl)-2-oxoethyl 4-aminobenzoate

Space Group:	P212121	***Cell:***	***a***	5.250(1)	***b***	8.271(1)	***c***	32.035(6)
Space Group No.:	19	***(Å,°)***	α	90.00	β	90.00	γ	90.00
R-Factor (%):	5.62	***Temperature(K):***	296	***Density(g/cm³):***	1.596			

Parameters

Fragment 1
ANG1 (A) 75.973
ANG2 (A) 57.749
ANG3 (A) 53.378

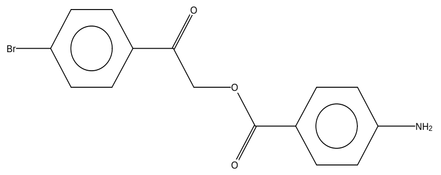

CIYFUN

Reference:	C.S.C.Kumar, Tze Shyang Chia, S.Chandraju, Chin Wei Ooi, Ching Kheng Quah, Hoong-Kun Fun (2014) *Z.Krist.Cryst.Mater.* ,**229,**328
Formula:	C_{15} H_{11} Br_1 O_3
Compound Name:	2-(4-bromophenyl)-2-oxoethyl benzoate

Space Group:	P21/c	***Cell:***	***a***	8.393(0)	***b***	10.458(1)	***c***	31.286(3)
Space Group No.:	14	***(Å,°)***	α	90.00	β	92.53(0)	γ	90.00
R-Factor (%):	5.35	***Temperature(K):***	296	***Density(g/cm³):***	1.545			

Parameters

Fragment 1
ANG1 (A) 68.699
ANG2 (A) 44.352
ANG3 (A) 53.624

Fragment 2
ANG1 (A) 72.430
ANG2 (A) 52.078
ANG3 (A) 46.758

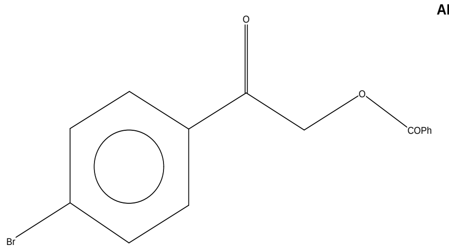

CIYGAU

Reference:	C.S.C.Kumar, Tze Shyang Chia, S.Chandraju, Chin Wei Ooi, Ching Kheng Quah, Hoong-Kun Fun (2014) *Z.Krist.Cryst.Mater.* ,**229,**328
Formula:	C_{15} H_{10} Br_1 Cl_1 O_3
Compound Name:	2-(4-bromophenyl)-2-oxoethyl 2-chlorobenzoate

Space Group:	Pbca	***Cell:***	***a***	12.326(1)	***b***	8.353(0)	***c***	27.305(3)
Space Group No.:	61	***(Å,°)***	α	90.00	β	90.00	γ	90.00
R-Factor (%):	3.79	***Temperature(K):***	296	***Density(g/cm³):***	1.671			

Parameters

Fragment 1
ANG1 (A) 6.555
ANG2 (A) 5.832
ANG3 (A) 1.619

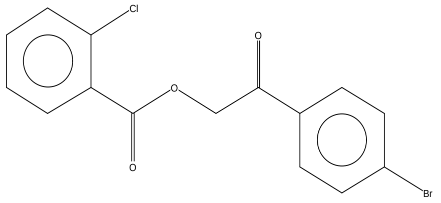

EVAFOX

Reference:	H.-K.Fun, S.Arshad, B.Garudachari, A.M.Isloor, M.N.Satyanarayan (2011) *Acta Crystallogr.,Sect.E:Struct.Rep.Online ,* **67,**o1582
Formula:	C_{15} H_{10} Br_2 O_3
Compound Name:	2-(4-Bromophenyl)-2-oxoethyl 4-bromobenzoate

Space Group:	Pc	***Cell:***	***a***	11.048(0)	***b***	5.908(0)	***c***	33.855(0)
Space Group No.:	7	***(Å,°)***	α	90.00	β	108.80(0)	γ	90.00
R-Factor (%):	4.73	***Temperature(K):***	100	***Density(g/cm³):***	1.896			

Parameters

Fragment 1
ANG1 (A) 3.157
ANG2 (A) 3.347
ANG3 (A) 4.064

Fragment 2
ANG1 (A) 54.590
ANG2 (A) 33.377
ANG3 (A) 53.965

Fragment 3
ANG1 (A) 6.081
ANG2 (A) 5.580
ANG3 (A) 6.130

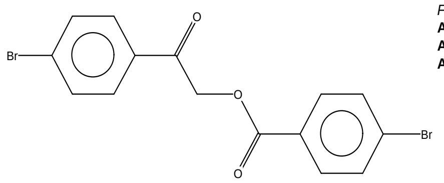

Search: search1 (Sun Oct 13 16:24:06 2019): Hits 29-32

EVAJAN

Reference: H.-K.Fun, W.-S.Loh, B.Garudachari, A.M.Isloor, M.N.Satyanarayan (2011) *Acta Crystallogr.,Sect.E:Struct.Rep.Online* , **67,**o1597

Formula: $C_{16}\,H_{10}\,Cl_1\,F_3\,O_3$

Compound Name: 2-(4-Chlorophenyl)-2-oxoethyl 3-(trifluoromethyl)benzoate

Space Group:	P21/c	***Cell:***	***a*** 14.304(0)	***b*** 12.133(0)	***c*** 8.546(0)		
Space Group No.:	14	***(Å,°)***	α 90.00	β 101.44(0)	γ 90.00		
R-Factor (%):	4.83	***Temperature(K):***	100	***Density(g/cm³):***	1.566		

Parameters

Fragment 1
ANG1 (A) 15.498
ANG2 (A) 3.575
ANG3 (A) 14.427

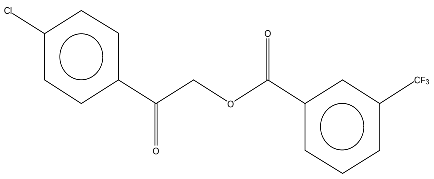

EVAJIV

Reference: H.-K.Fun, S.Arshad, B.Garudachari, A.M.Isloor, M.N.Satyanarayan (2011) *Acta Crystallogr.,Sect.E:Struct.Rep.Online* , **67,**o1599

Formula: $C_{15}\,H_9\,Cl_1\,F_2\,O_3$

Compound Name: 2-(4-Chlorophenyl)-2-oxoethyl 2,4-difluorobenzoate

Space Group:	P21/c	***Cell:***	***a*** 16.018(1)	***b*** 7.961(0)	***c*** 24.017(1)
Space Group No.:	14	***(Å,°)***	α 90.00	β 115.94(0)	γ 90.00
R-Factor (%):	5.42	***Temperature(K):***	296	***Density(g/cm³):***	1.499

Parameters

Fragment 1
ANG1 (A) 7.922
ANG2 (A) 19.331
ANG3 (A) 12.874

Fragment 2
ANG1 (A) 73.504
ANG2 (A) 49.205
ANG3 (A) 60.714

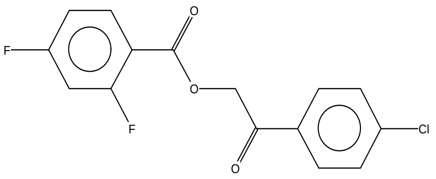

EVAZEH

Reference: H.-K.Fun, S.I.J.Asik, B.Garudachari, A.M.Isloor, M.N.Satyanarayan (2011) *Acta Crystallogr.,Sect.E:Struct.Rep.Online* , **67,**o1687

Formula: $C_{16}\,H_{13}\,Cl_1\,O_4$

Compound Name: 2-(4-Chlorophenyl)-2-oxoethyl 2-methoxybenzoate

Space Group:	Pbca	***Cell:***	***a*** 7.721(0)	***b*** 14.441(1)	***c*** 26.064(2)
Space Group No.:	61	***(Å,°)***	α 90.00	β 90.00	γ 90.00
R-Factor (%):	4.10	***Temperature(K):***	296	***Density(g/cm³):***	1.393

Parameters

Fragment 1
ANG1 (A) 86.378
ANG2 (A) 47.391
ANG3 (A) 75.422

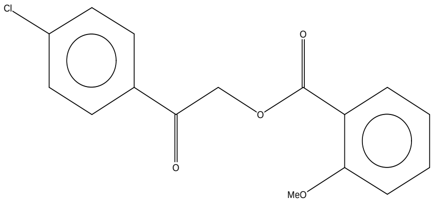

EVEGIW

Reference: H.-K.Fun, C.S.Yeap, B.Garudachari, A.M.Isloor, M.N.Satyanarayan (2011) *Acta Crystallogr.,Sect.E:Struct.Rep.Online* , **67,**o1723

Formula: $C_{15}\,H_{10}\,Br_1\,Cl_1\,O_3$

Compound Name: 2-(4-Bromophenyl)-2-oxoethyl 4-chlorobenzoate

Space Group:	P21	***Cell:***	***a*** 17.106(1)	***b*** 5.306(0)	***c*** 24.038(1)
Space Group No.:	4	***(Å,°)***	α 90.00	β 101.50(0)	γ 90.00
R-Factor (%):	4.49	***Temperature(K):***	297	***Density(g/cm³):***	1.648

Parameters

Fragment 1
ANG1 (A) 0.745
ANG2 (A) 3.455
ANG3 (A) 3.889

Fragment 2
ANG1 (A) 66.087
ANG2 (A) 49.602
ANG3 (A) 52.536

Fragment 3
ANG1 (A) 68.779
ANG2 (A) 49.032
ANG3 (A) 54.911

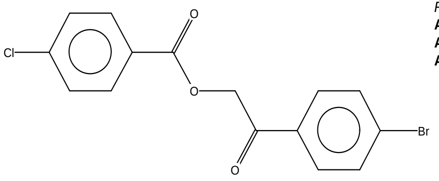

Search: search1 (Sun Oct 13 16:24:06 2019): Hits 33-36

EVEGOC

Reference: H.-K.Fun, C.K.Quah, B.Garudachari, A.M.Isloor, M.N.Satyanarayan (2011) *Acta Crystallogr.,Sect.E:Struct.Rep.Online* , **67,**o1724

Formula: $C_{16}\ H_{13}\ Br_1\ O_4$

Compound Name: 2-(4-Bromophenyl)-2-oxoethyl 2-methoxybenzoate

Space Group: Pbca ***Cell:*** ***a*** 7.842(0) ***b*** 14.680(0) ***c*** 25.768(1)
Space Group No.: 61 ***(Å,°)*** α 90.00 β 90.00 γ 90.00

R-Factor (%): 3.68 ***Temperature(K):*** 296 ***Density(g/cm^3):*** 1.564

Parameters

Fragment 1
ANG1 (A) 85.921
ANG2 (A) 48.705
ANG3 (A) 74.044

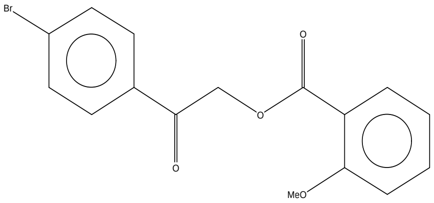

EVEVEH

Reference: H.-K.Fun, T.Shahani, B.Garudachari, A.M.Isloor, M.N.Satyganarayan (2011) *Acta Crystallogr.,Sect.E:Struct.Rep.Online* , **67,**o1802

Formula: $C_{15}\ H_{11}\ Cl_1\ O_3$

Compound Name: 2-(4-Chlorophenyl)-2-oxoethyl benzoate

Space Group: P21/c ***Cell:*** ***a*** 8.196(0) ***b*** 10.872(1) ***c*** 16.542(1)
Space Group No.: 14 ***(Å,°)*** α 90.00 β 117.82(0) γ 90.00

R-Factor (%): 4.41 ***Temperature(K):*** 296 ***Density(g/cm^3):*** 1.400

Parameters

Fragment 1
ANG1 (A) 84.286
ANG2 (A) 50.331
ANG3 (A) 65.552

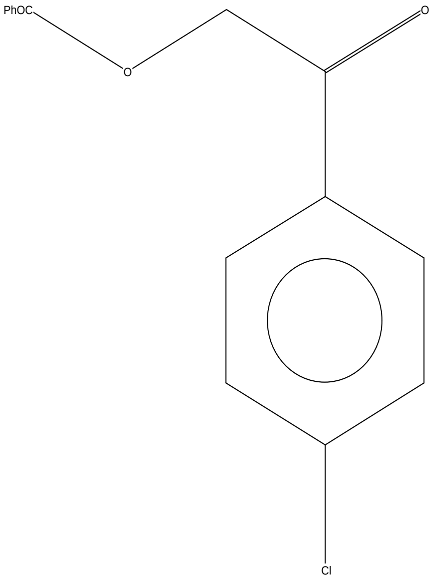

GARCEJ

Reference: A.M.Isloor, B.Garudachari, M.N.Satyanarayan, T.Gerber, E.Hosten, R.Betz (2012) *Acta Crystallogr.,Sect.E:Struct.Rep.Online* , **68,**o513

Formula: $C_{16}\ H_{13}\ F_1\ O_4$

Compound Name: 2-(4-Fluorophenyl)-2-oxoethyl 2-methoxybenzoate

Space Group: P21/c ***Cell:*** ***a*** 7.937(0) ***b*** 26.446(0) ***c*** 7.064(0)
Space Group No.: 14 ***(Å,°)*** α 90.00 β 113.40(0) γ 90.00

R-Factor (%): 3.73 ***Temperature(K):*** 200 ***Density(g/cm^3):*** 1.407

Parameters

Fragment 1
ANG1 (A) 73.676
ANG2 (A) 45.791
ANG3 (A) 73.072

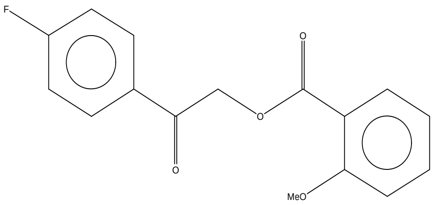

GESBUE

Reference: Huirong Zhang, Anuj Kunadia, Yingfu Lin, J.D.Fondell, D.Seidel, Huizhou Fan (2017) *Plos One* ,**12,**e0185783

Formula: $C_{21}\ H_{17}\ N_1\ O_3$

Compound Name: 2-(biphenyl-4-yl)-2-oxoethyl 3-aminobenzoate

Space Group: P-1 ***Cell:*** ***a*** 5.293(0) ***b*** 11.682(1) ***c*** 13.404(1)
Space Group No.: 2 ***(Å,°)*** α 87.86(0) β 83.98(0) γ 87.70(0)

R-Factor (%): 5.45 ***Temperature(K):*** 294 ***Density(g/cm^3):*** 1.337

Parameters

Fragment 1
ANG1 (A) 75.690
ANG2 (A) 45.492
ANG3 (A) 61.521

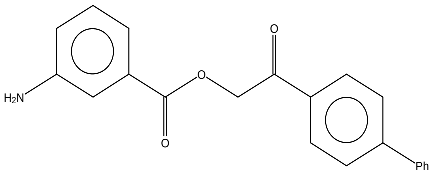

Search: search1 (Sun Oct 13 16:24:06 2019): Hits 37-40

GITHUN

Reference: Yi Jin, Jian-Nan Guo, Kan Lin, Guo Tang, Yu-Fen Zhao (2008) *Acta Crystallogr.,Sect.E:Struct.Rep.Online* ,**64,**o507

Formula: $C_{15}\,H_{11}\,Cl_{1}\,O_{3}$

Compound Name: Benzoylmethyl 4-chlorobenzoate

Space Group: P21/c
Space Group No.: 14

Cell: ***(Å,°)*** ***a*** 14.763(0) ***b*** 16.451(0) ***c*** 15.821(0) α 90.00 β 92.11(0) γ 90.00

R-Factor (%): 3.65 ***Temperature(K):*** 293 ***Density(g/cm³):*** 1.425

Parameters

Fragment 1
ANG1 (A) 77.288
ANG2 (A) 43.515
ANG3 (A) 66.107

Fragment 2
ANG1 (A) 61.687
ANG2 (A) 36.913
ANG3 (A) 58.896

Fragment 3
ANG1 (A) 77.666
ANG2 (A) 47.925
ANG3 (A) 73.608

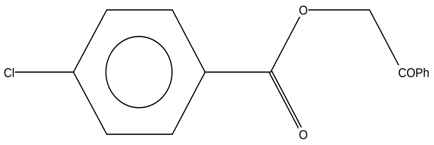

IDIWID

Reference: B.Garudachari, A.M.Isloor, T.Gerber, E.Hosten, R.Betz (2013) *Acta Crystallogr.,Sect.E:Struct.Rep.Online* ,**69,**o649

Formula: $C_{19}\,H_{13}\,Br_{1}\,O_{3}$

Compound Name: 2-(4-Bromophenyl)-2-oxoethyl naphthalene-1-carboxylate

Synonym: 2-(4-Bromophenyl)-2-oxoethyl 1-naphthoate

Space Group: P21/c
Space Group No.: 14

Cell: ***(Å,°)*** ***a*** 5.289(0) ***b*** 14.914(0) ***c*** 20.027(0) α 90.00 β 100.87(0) γ 90.00

R-Factor (%): 3.40 ***Temperature(K):*** 200 ***Density(g/cm³):*** 1.581

Parameters

Fragment 1
ANG1 (A) 78.182
ANG2 (A) 60.487
ANG3 (A) 84.596

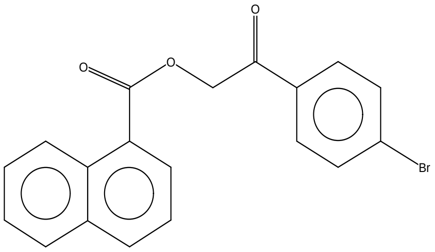

KULLIO

Reference: Diwaker, C.S.Chidan Kumar, A.Kumar, S.Chandraju, Hoong-Kun Fun, Ching Kheng Quah (2015) *J.Mol.Struct.* ,**1092,**192

Formula: $C_{16}\,H_{13}\,Br_{1}\,O_{3}$

Compound Name: 2-(4-bromophenyl)-2-oxoethyl 3-methylbenzoate

Space Group: P21/c
Space Group No.: 14

Cell: ***(Å,°)*** ***a*** 8.176(2) ***b*** 7.828(2) ***c*** 22.952(6) α 90.00 β 91.33(0) γ 90.00

R-Factor (%): 4.48 ***Temperature(K):*** 296 ***Density(g/cm³):*** 1.507

Parameters

Fragment 1
ANG1 (A) 86.034
ANG2 (A) 57.191
ANG3 (A) 74.641

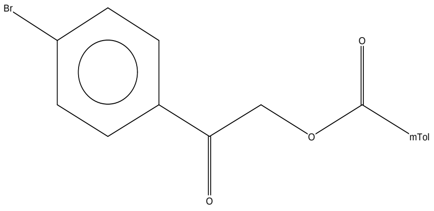

MANGIR

Reference: I.V.Komarov, M.V.Gorichko, O.V.Shishkin, M.Yu.Kornilov (1999) *Zh.Org.Khim.(Russ.)(Russ.J.Org.Chem.)* ,**35,**1416

Formula: $C_{18}\,H_{17}\,Br_{1}\,O_{3}$

Compound Name: Phenacyl 5-bromo-2,3,4-trimethylbenzoate

Space Group: P-1
Space Group No.: 2

Cell: ***(Å,°)*** ***a*** 10.200(6) ***b*** 10.338(4) ***c*** 16.110(7) α 90.63(1) β 93.11(1) γ 97.49(1)

R-Factor (%): 7.07 ***Temperature(K):*** 295 ***Density(g/cm³):*** 1.427

Parameters

Fragment 1
ANG1 (A) 70.746
ANG2 (A) 61.014
ANG3 (A) 60.463

Fragment 2
ANG1 (A) 77.848
ANG2 (A) 57.899
ANG3 (A) 67.499

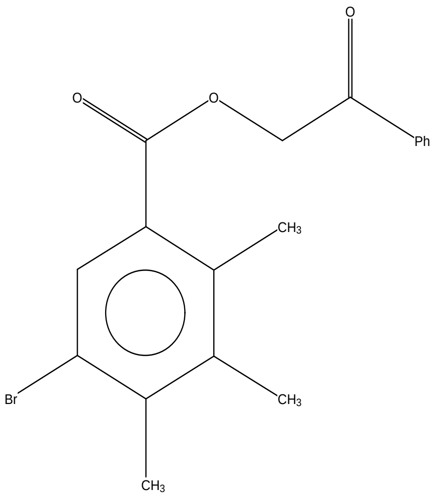

Search: search1 (Sun Oct 13 16:24:06 2019): Hits 41-44

OBOYIP

Reference: H.-K.Fun, T.Shahani, B.Garudachari, A.M.Isloor, M.N.Satyanarayan (2011) *Acta Crystallogr.,Sect.E:Struct.Rep.Online* , **67,**o3154

Formula: $C_{16} H_{13} Br_1 O_3$

Compound Name: 2-(4-Bromophenyl)-2-oxoethyl 4-methylbenzoate

Space Group: P21/c	***Cell:***	***a*** 5.837(0)	***b*** 8.344(0)	***c*** 27.968(0)
Space Group No.: 14	***(Å,°)***	α 90.00	β 95.18(0)	γ 90.00

R-Factor (%): 2.81 ***Temperature(K):*** 100 ***Density(g/cm^3):*** 1.631

Parameters

Fragment 1
ANG1 (A) 80.594
ANG2 (A) 42.955
ANG3 (A) 69.145

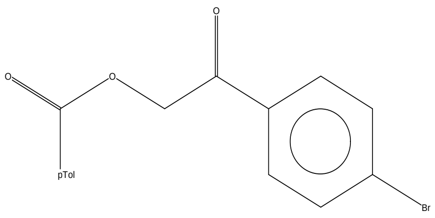

OCAKUA

Reference: H.-K.Fun, C.K.Quah, A.M.Vijesh, A.M.Isloor, T.Arulmoli (2011) *Acta Crystallogr.,Sect.E:Struct.Rep.Online* ,**67,**o3351

Formula: $C_{17} H_{15} Cl_1 O_5$

Compound Name: 2-(4-Chlorophenyl)-2-oxoethyl 3,4-dimethoxybenzoate

Space Group: P-1	***Cell:***	***a*** 8.228(0)	***b*** 9.338(0)	***c*** 10.599(0)
Space Group No.: 2	***(Å,°)***	α 89.06(0)	β 76.75(0)	γ 83.67(0)

R-Factor (%): 5.20 ***Temperature(K):*** 296 ***Density(g/cm^3):*** 1.411

Parameters

Fragment 1
ANG1 (A) 74.449
ANG2 (A) 52.177
ANG3 (A) 64.990

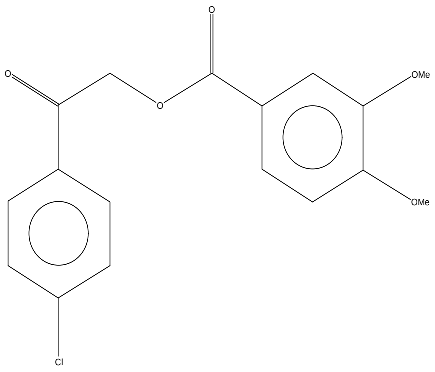

OCAQUG

Reference: H.-K.Fun, T.S.Chia, S.Shenvi, A.M.Isloor, B.Garudachari (2011) *Acta Crystallogr.,Sect.E:Struct.Rep.Online* ,**67,**o3379

Formula: $C_{16} H_{12} Cl_2 O_4$

Compound Name: 2-(2,4-Dichlorophenyl)-2-oxoethyl 4-methoxybenzoate

Space Group: P21/c	***Cell:***	***a*** 9.051(0)	***b*** 7.085(0)	***c*** 23.334(0)
Space Group No.: 14	***(Å,°)***	α 90.00	β 102.51(0)	γ 90.00

R-Factor (%): 3.89 ***Temperature(K):*** 100 ***Density(g/cm^3):*** 1.542

Parameters

Fragment 1
ANG1 (A) 70.108
ANG2 (A) 49.241
ANG3 (A) 80.999

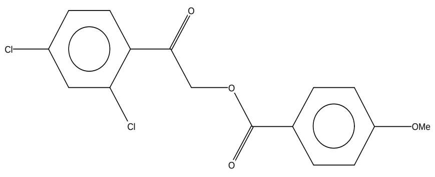

OCEFEJ

Reference: H.-K.Fun, W.-S.Loh, B.Garudachari, A.M.Isloor, M.N.Satyanarayan (2011) *Acta Crystallogr.,Sect.E:Struct.Rep.Online* , **67,**o3456

Formula: $C_{16} H_{13} F_1 O_4$

Compound Name: 2-(4-Fluorophenyl)-2-oxoethyl 4-methoxybenzoate

Space Group: P21/c	***Cell:***	***a*** 9.352(0)	***b*** 10.195(0)	***c*** 15.647(0)
Space Group No.: 14	***(Å,°)***	α 90.00	β 118.84(0)	γ 90.00

R-Factor (%): 5.49 ***Temperature(K):*** 100 ***Density(g/cm^3):*** 1.465

Parameters

Fragment 1
ANG1 (A) 84.275
ANG2 (A) 44.601
ANG3 (A) 80.665

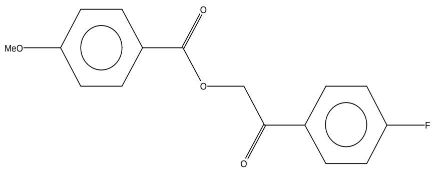

Search: search1 (Sun Oct 13 16:24:06 2019): Hits 45-48

PAXCOI

Reference: H.-K.Fun, S.I.J.Asik, B.Garudachari, A.M.Isloor, M.Satyanarayan (2012) *Acta Crystallogr.,Sect.E:Struct.Rep.Online* ,**68,** o1876

Formula: $C_{23} H_{15} Br_1 O_3$

Compound Name: 2-(4-Bromophenyl)-2-oxoethyl anthracene-9-carboxylate

Space Group:	P21/c	***Cell:***	***a*** 10.191(0)	***b*** 15.059(1)	***c*** 13.794(0)
Space Group No.:	14	***(Å,°)***	α 90.00	β 122.38(0)	γ 90.00

R-Factor (%): 4.52 ***Temperature(K):*** 100 ***Density(g/cm³):*** 1.558

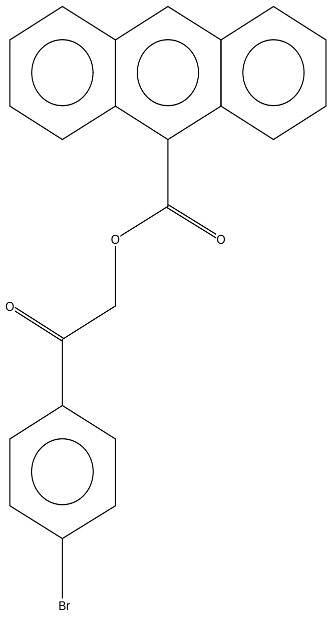

Parameters

Fragment 1
ANG1 (A) 5.499
ANG2 (A) 59.667
ANG3 (A) 63.073

PECZAA

Reference: I.Khan, A.Ibrar, A.Korzanski, M.Kubicki (2012) *Acta Crystallogr.,Sect.E:Struct.Rep.Online* ,**68,**o3465

Formula: $C_{16} H_{13} Br_1 O_3$

Compound Name: 2-(4-Methylphenyl)-2-oxoethyl 3-bromobenzoate

Space Group:	P-1	***Cell:***	***a*** 4.798(0)	***b*** 10.995(0)	***c*** 14.165(0)
Space Group No.:	2	***(Å,°)***	α 74.83(0)	β 87.76(0)	γ 79.33(0)

R-Factor (%): 3.78 ***Temperature(K):*** 295 ***Density(g/cm³):*** 1.561

Parameters

Fragment 1
ANG1 (A) 46.173
ANG2 (A) 19.889
ANG3 (A) 27.092

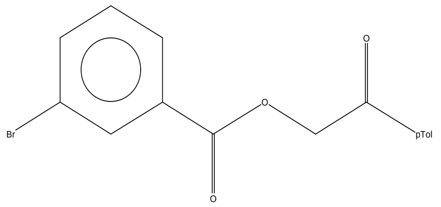

PODQIK

Reference: C.S.Chidan Kumar, C.Yohannan Panicker, Hoong-Kun Fun, Y.Sheena Mary, B.Harikumar, S.Chandraju, Ching Kheng Quah, Chin Wei Ooi (2014) *Spectrochim.Acta,Part A* , **128,**327

Formula: $C_{16} H_{13} Cl_1 O_3$

Compound Name: 2-(4-chlorophenyl)-2-oxoethyl 3-methylbenzoate

Space Group:	P21/c	***Cell:***	***a*** 4.744(0)	***b*** 29.316(5)	***c*** 10.421(1)
Space Group No.:	14	***(Å,°)***	α 90.00	β 92.76(0)	γ 90.00

R-Factor (%): 4.90 ***Temperature(K):*** 297 ***Density(g/cm³):*** 1.325

Parameters

Fragment 1
ANG1 (A) 67.811
ANG2 (A) 51.421
ANG3 (A) 80.526

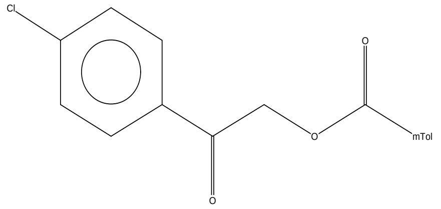

PODRAD

Reference: C.S.Chidan Kumar, C.Yohannan Panicker, Hoong-Kun Fun, Y.Sheena Mary, B.Harikumar, S.Chandraju, Ching Kheng Quah, Chin Wei Ooi (2014) *Spectrochim.Acta,Part A* , **126,**208

Formula: $C_{15} H_{10} Cl_1 N_1 O_5$

Compound Name: 2-(4-chlorophenyl)-2-oxoethyl 3-nitrobenzoate

Space Group:	P-1	***Cell:***	***a*** 9.258(1)	***b*** 12.106(1)	***c*** 14.123(1)
Space Group No.:	2	***(Å,°)***	α 100.79(0)	β 100.20(1)	γ 106.21(0)

R-Factor (%): 5.60 ***Temperature(K):*** 297 ***Density(g/cm³):*** 1.466

Parameters

Fragment 1
ANG1 (A) 3.702
ANG2 (A) 2.438
ANG3 (A) 3.207

Fragment 2
ANG1 (A) 5.918
ANG2 (A) 3.974
ANG3 (A) 2.999

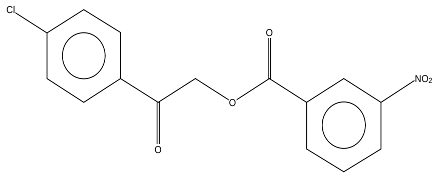

Search: search1 (Sun Oct 13 16:24:06 2019): Hits 49-52

QIKVAH

Reference: Y.Kashiwada, K.Yamazaki, Y.Ikeshiro, T.Yamagishi, T.Fujioka, K.Mihashi, K.Mizuki, L.M.Cosentino, K.Fowke, S.L.Morris-Natschke, Kuo-Hsiung Lee (2001) *Tetrahedron* ,**57,**1559

Formula: $C_{31} H_{35} Br_1 O_5$

Compound Name: p-Bromophenacyl rhododaurichromanic acid A

Space Group:	P21	***Cell:***	***a*** 8.505(2)	***b*** 27.809(0)	***c*** 5.901(1)
Space Group No.:	4	***(Å,°)***	α 90.00	β 95.32(2)	γ 90.00

R-Factor (%): 3.59 ***Temperature(K):*** 295 ***Density(g/cm³):*** 1.356

Parameters

Fragment 1
ANG1 (A) 89.757
ANG2 (A) 50.849
ANG3 (A) 76.771

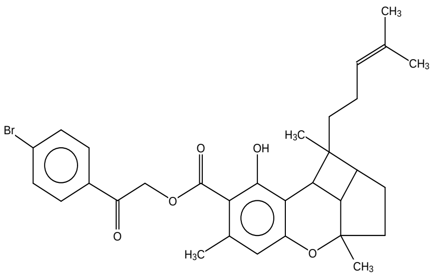

USIWID

Reference: H.-K.Fun, S.Arshad, B.Garudachari, A.M.Isloor, M.N.Satyanarayan (2011) *Acta Crystallogr.,Sect.E:Struct.Rep.Online* , **67,**o1528

Formula: $C_{15} H_{12} O_3$

Compound Name: 2-Oxo-2-phenylethyl benzoate

Space Group:	P21/c	***Cell:***	***a*** 9.030(1)	***b*** 14.116(2)	***c*** 9.638(1)
Space Group No.:	14	***(Å,°)***	α 90.00	β 90.56(0)	γ 90.00

R-Factor (%): 5.97 ***Temperature(K):*** 296 ***Density(g/cm³):*** 1.299

Parameters

Fragment 1
ANG1 (A) 86.092
ANG2 (A) 67.451
ANG3 (A) 56.918

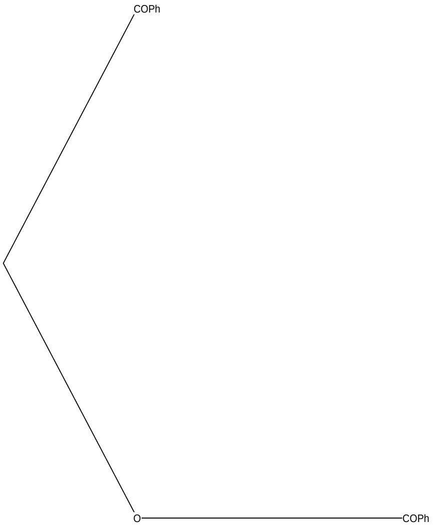

USIWID01

Reference: S.Khamarui, R.Maiti, D.K.Maiti (2015) *Chem.Commun.* ,**51,** 384

Formula: $C_{15} H_{12} O_3$

Compound Name: 2-Oxo-2-phenylethyl benzoate

Space Group:	P21/c	***Cell:***	***a*** 9.016(1)	***b*** 14.112(2)	***c*** 9.680(1)
Space Group No.:	14	***(Å,°)***	α 90.00	β 90.55(0)	γ 90.00

R-Factor (%): 6.96 ***Temperature(K):*** 296 ***Density(g/cm³):*** 1.296

Parameters

Fragment 1
ANG1 (A) 85.431
ANG2 (A) 67.111
ANG3 (A) 56.679

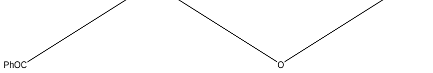

USIWID02

Reference: Meng-Yang Chang (2016) *CSD Communication(Private Communication)* ,

Formula: $C_{15} H_{12} O_3$

Compound Name: 2-oxo-2-phenylethyl benzoate

Space Group:	P21/c	***Cell:***	***a*** 9.067(0)	***b*** 14.127(0)	***c*** 9.608(0)
Space Group No.:	14	***(Å,°)***	α 90.00	β 90.78(0)	γ 90.00

R-Factor (%): 4.46 ***Temperature(K):*** 296 ***Density(g/cm³):*** 1.297

Parameters

Fragment 1
ANG1 (A) 86.592
ANG2 (A) 67.891
ANG3 (A) 57.034

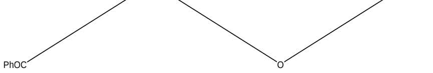

Search: search1 (Sun Oct 13 16:24:06 2019): Hits 53-56

USIWOJ

Reference:	H.-K.Fun, W.-S.Loh, B.Garudachari, A.M.Isloor, M.N.Satyanarayan (2011) *Acta Crystallogr.,Sect.E:Struct.Rep.Online* , **67,**o1529
Formula:	$C_{16} H_{13} Br_1 O_4$
Compound Name:	2-(4-Bromophenyl)-2-oxoethyl 4-methoxybenzoate

Space Group:	P-1	***Cell:***	***a*** 7.970(0)	***b*** 7.985(0)	***c*** 11.319(0)		
Space Group No.:	2	***(Å,°)***	α 86.54(0)	β 83.20(0)	γ 89.63(0)		
R-Factor (%):	2.65	***Temperature(K):***	296	***Density(g/cm³):***	1.624		

Parameters

Fragment 1

**ANG1 (A)**	84.066
**ANG2 (A)**	58.224
**ANG3 (A)**	80.862

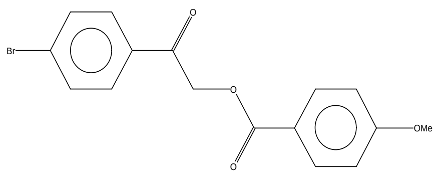

VOBYUI

Reference:	C.S.Chidan Kumar, Hoong Kun Fun, M.Tursun, Chin Wei Ooi, S.Chandraju, Ching Kheng Quah, C.Parlak (2014) *Spectrochim.Acta,Part A* ,**124,**595
Formula:	$C_{15} H_{10} Cl_2 O_3$
Compound Name:	2-(4-Chlorophenyl)-2-oxoethyl 2-chlorobenzoate

Space Group:	Pbca	***Cell:***	***a*** 12.312(5)	***b*** 8.103(3)	***c*** 27.565(11)		
Space Group No.:	61	***(Å,°)***	α 90.00	β 90.00	γ 90.00		
R-Factor (%):	8.71	***Temperature(K):***	293	***Density(g/cm³):***	1.493		

Parameters

Fragment 1

**ANG1 (A)**	5.954
**ANG2 (A)**	5.773
**ANG3 (A)**	1.689

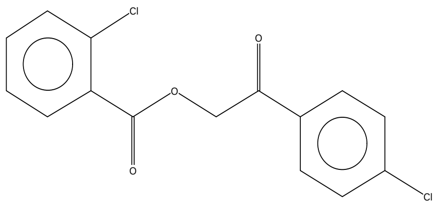

WIGTUD

Reference:	B.Garudachari, A.M.Isloor, T.Gerber, E.Hosten, R.Betz (2013) *Acta Crystallogr.,Sect.E:Struct.Rep.Online* ,**69,**o0551
Formula:	$C_{19} H_{13} Cl_1 O_3$
Compound Name:	2-(4-Chlorophenyl)-2-oxoethyl naphthalene-1-carboxylate
Synonym:	2-(4-Chlorophenyl)-2-oxoethyl 1-naphthoate

Space Group:	P21/c	***Cell:***	***a*** 5.271(0)	***b*** 14.847(0)	***c*** 19.843(0)		
Space Group No.:	14	***(Å,°)***	α 90.00	β 100.38(0)	γ 90.00		
R-Factor (%):	3.54	***Temperature(K):***	200	***Density(g/cm³):***	1.412		

Parameters

Fragment 1

**ANG1 (A)**	78.056
**ANG2 (A)**	60.249
**ANG3 (A)**	84.496

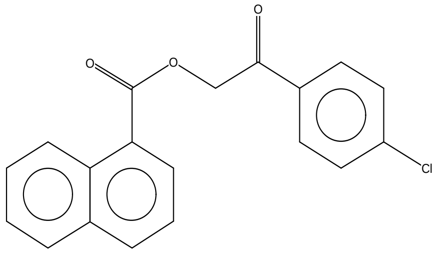

YAFWEJ

Reference:	H.-K.Fun, S.Arshad, B.Garudachari, A.M.Isloor, K.N.Shivananda (2011) *Acta Crystallogr.,Sect.E:Struct.Rep.Online* ,**67,** o2836
Formula:	$C_{16} H_{10} F_4 O_3$
Compound Name:	2-(4-Fluorophenyl)-2-oxoethyl 3-(trifluoromethyl)benzoate

Space Group:	P21/c	***Cell:***	***a*** 14.769(1)	***b*** 12.160(1)	***c*** 8.093(1)		
Space Group No.:	14	***(Å,°)***	α 90.00	β 95.89(0)	γ 90.00		
R-Factor (%):	5.74	***Temperature(K):***	296	***Density(g/cm³):***	1.499		

Parameters

Fragment 1

**ANG1 (A)**	20.336
**ANG2 (A)**	5.349
**ANG3 (A)**	17.215

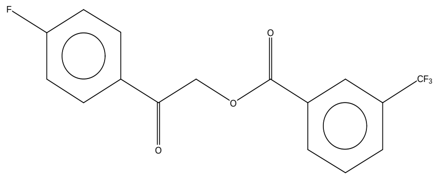

Search: search1 (Sun Oct 13 16:24:06 2019): Hits 57-60

YAFZAI

Reference: H.-K.Fun, W.-S.Loh, B.Garudachari, A.M.Isloor, M.N.Satyanarayana (2011) *Acta Crystallogr.,Sect.E:Struct.Rep.Online* , **67,**o2854

Formula: $C_{15}\ H_{11}\ Br_1\ O_4$

Compound Name: 2-(4-Bromophenyl)-2-oxoethyl 4-hydroxybenzoate

Space Group:	P21/c	***Cell:***	***a***	6.292(0)	***b***	7.789(0)	***c***	26.750(0)
Space Group No.:	14	***(Å,°)***	α	90.00	β	98.23(0)	γ	90.00

R-Factor (%): 3.36 ***Temperature(K):*** 100 ***Density(g/cm^3):*** 1.716

Parameters

Fragment 1
ANG1 (A) 66.766
ANG2 (A) 44.992
ANG3 (A) 56.390

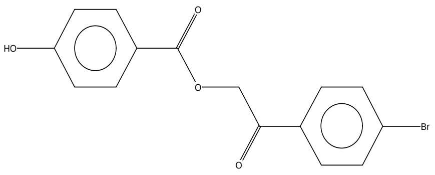

YAHGUL

Reference: H.-K.Fun, W.-S.Loh, B.Garudachari, A.M.Isloor, M.N.Satyanarayana (2011) *Acta Crystallogr.,Sect.E:Struct.Rep.Online* , **67,**o3030

Formula: $C_{16}\ H_{13}\ Cl_1\ O_3$

Compound Name: 2-(4-Chlorophenyl)-2-oxoethyl 4-methylbenzoate

Space Group:	P21/c	***Cell:***	***a***	5.913(0)	***b***	8.504(0)	***c***	27.877(1)
Space Group No.:	14	***(Å,°)***	α	90.00	β	95.88(0)	γ	90.00

R-Factor (%): 4.60 ***Temperature(K):*** 297 ***Density(g/cm^3):*** 1.375

Parameters

Fragment 1
ANG1 (A) 80.735
ANG2 (A) 44.446
ANG3 (A) 68.625

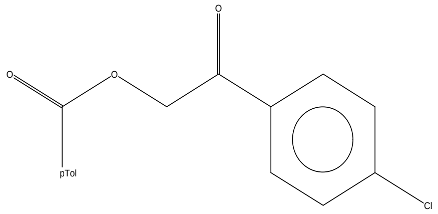

YAHYOX

Reference: H.-K.Fun, C.W.Ooi, B.Garudachari, A.M.Isloor, M.N.Satyanarayan (2011) *Acta Crystallogr.,Sect.E:Struct.Rep.Online* , **67,**o3119

Formula: $C_{16}\ H_{13}\ Br_1\ O_3$

Compound Name: 2-(4-Bromophenyl)-2-oxoethyl 2-methylbenzoate

Space Group:	P21/c	***Cell:***	***a***	5.452(0)	***b***	31.238(0)	***c***	9.721(0)
Space Group No.:	14	***(Å,°)***	α	90.00	β	120.41(0)	γ	90.00

R-Factor (%): 4.01 ***Temperature(K):*** 100 ***Density(g/cm^3):*** 1.550

Parameters

Fragment 1
ANG1 (A) 66.659
ANG2 (A) 52.766
ANG3 (A) 87.307

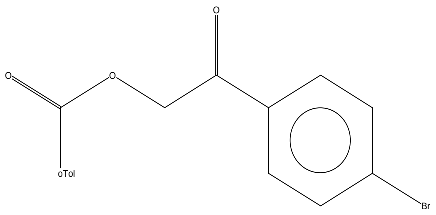

ZARCED

Reference: S.Kumar, Chandra, B.M.Rajesh, M.Mahendra, B.H.Doreswamy (2017) *IUCrData* ,**2,**x170183

Formula: $C_{18}\ H_{18}\ O_5$

Compound Name: 2-(4-methylphenyl)-2-oxoethyl 3,4-dimethoxybenzoate

Space Group:	P-1	***Cell:***	***a***	7.981(0)	***b***	8.609(0)	***c***	11.467(0)
Space Group No.:	2	***(Å,°)***	α	99.68(0)	β	99.17(0)	γ	91.50(0)

R-Factor (%): 4.57 ***Temperature(K):*** 293 ***Density(g/cm^3):*** 1.364

Parameters

Fragment 1
ANG1 (A) 66.545
ANG2 (A) 42.982
ANG3 (A) 87.728

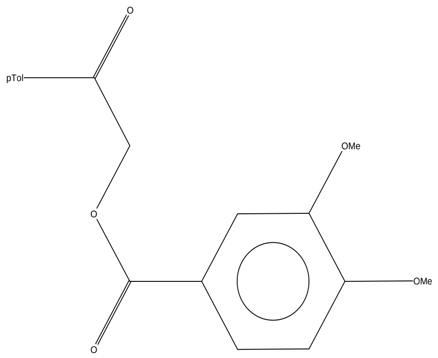

Search: search1 (Sun Oct 13 16:24:06 2019): Hits 61-62

POCGAS

Reference: Taoshan Xu, Chaoxian Yan, Yuewei Wu, Chengshan Yuan, Xiangfeng Shao (2019) *Dyes Pigm.* ,**168,**235

Formula: $C_{16} H_{12} Cl_2 O_4$

Compound Name: 2-(3,4-dichlorophenyl)-2-oxoethyl 3-methoxybenzoate

Space Group: P21/c
Space Group No.: 14

Cell: ***(Å,°)***			
	a 4.262(1)	***b*** 31.860(10)	***c*** 11.222(4)
	α 90.00	β 101.92(1)	γ 90.00

R-Factor (%): 5.84 ***Temperature(K):*** 296 ***Density(g/cm^3):*** 1.511

Parameters

Fragment 1
ANG1 (A) 37.702
ANG2 (A) 18.527
ANG3 (A) 19.405

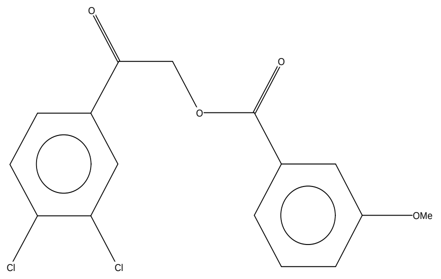

POCGEW

Reference: Taoshan Xu, Chaoxian Yan, Yuewei Wu, Chengshan Yuan, Xiangfeng Shao (2019) *Dyes Pigm.* ,**168,**235

Formula: $C_{16} H_{12} Cl_2 O_4$

Compound Name: 2-(3,4-dichlorophenyl)-2-oxoethyl 4-methoxybenzoate

Space Group: Pbca
Space Group No.: 61

Cell: ***(Å,°)***			
	a 20.447(3)	***b*** 8.989(1)	***c*** 32.705(5)
	α 90.00	β 90.00	γ 90.00

R-Factor (%): 5.08 ***Temperature(K):*** 296 ***Density(g/cm^3):*** 1.499

Parameters

Fragment 1
ANG1 (A) 76.546
ANG2 (A) 51.387
ANG3 (A) 63.163

Fragment 2
ANG1 (A) 85.838
ANG2 (A) 57.137
ANG3 (A) 67.767

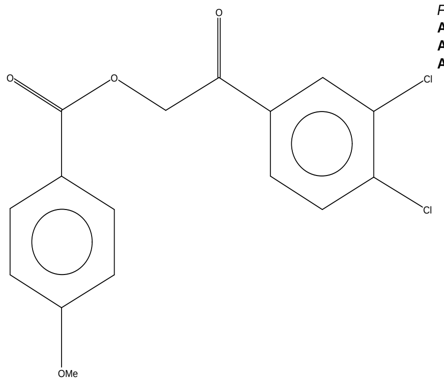

Supplement: Supplementary file 4 [file e-75-01719-sup3.pdf]
