# Supplementary material for: Crystal structure and Hirshfeld surface analysis of 2-(4-nitro­phen­yl)-2-oxoethyl benzoate
Source: Acta Crystallogr E Crystallogr Commun. 2019 Oct 22;75(Pt 11):1719–23. doi: 10.1107/S2056989019013975 (PMC6829730; doi:10.1107/S2056989019013975)

Search Overview

Search:	search2
Date/Time done:	Sun Oct 13 16:31:09 2019
Database(s):	CSD version 5.40 updates (Feb 2019)<br>CSD version 5.40 (November 2018)<br>CSD version 5.40 updates (May 2019)<br>CSD version 5.40 updates (Aug 2019)
Restriction Info:	No refcode restrictions applied
Filters:	None
Percentage Completed:	100%
Number of Hits:	7

Summary of queries used. Search found structures that:

match

Query 1 **Query 2**

Query 1

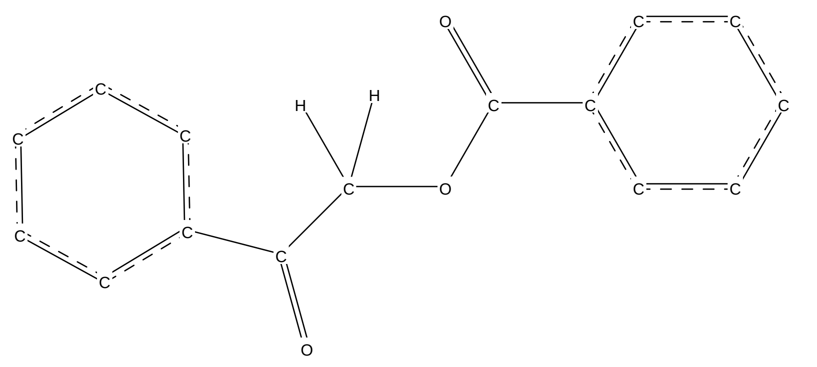

Query 2

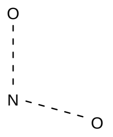

Search: search2 (Sun Oct 13 16:31:09 2019): Hits 1-4

CISRUU

Reference: Huey Chong Kwong, C.S.Chidan Kumar, Siau Hui Mah, Tze Shyang Chia, Ching Kheng Quah, Zi Han Loh, S.Chandraju, Gin Keat Lim (2017) *Plos One* ,**12,**e0170117

Formula: $C_{21}\ H_{15}\ N_1\ O_5$

Compound Name: 2-(biphenyl-4-yl)-2-oxoethyl 3-nitrobenzoate

Space Group:	P-1	***Cell:***	***a*** 4.933(0)		***b*** 18.312(1)		***c*** 19.167(1)
Space Group No.:	2	***(Å,°)***	α 103.22(0)		β 97.41(0)		γ 89.98(0)

R-Factor (%): 4.21 ***Temperature(K):*** 100 ***Density(g/cm³):*** 1.437

Parameters

Fragment 1
ANG1 (A) 64.060
ANG2 (A) 69.594
ANG3 (A) 65.756

Fragment 2
ANG1 (A) 64.087
ANG2 (A) 69.652
ANG3 (A) 65.704

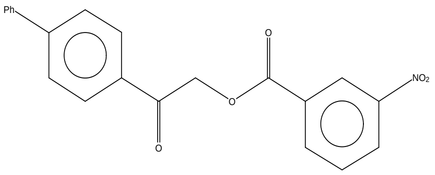

CISSAB

Reference: Huey Chong Kwong, C.S.Chidan Kumar, Siau Hui Mah, Tze Shyang Chia, Ching Kheng Quah, Zi Han Loh, S.Chandraju, Gin Keat Lim (2017) *Plos One* ,**12,**e0170117

Formula: $C_{21}\ H_{15}\ N_1\ O_5$

Compound Name: 2-(biphenyl-4-yl)-2-oxoethyl 4-nitrobenzoate

Space Group:	P21/c	***Cell:***	***a*** 8.681(1)	***b*** 5.555(0)	***c*** 35.401(5)
Space Group No.:	14	***(Å,°)***	α 90.00	β 90.12(0)	γ 90.00

R-Factor (%): 5.26 ***Temperature(K):*** 293 ***Density(g/cm³):*** 1.406

Parameters

Fragment 1
ANG1 (A) 70.959
ANG2 (A) 42.607
ANG3 (A) 82.524

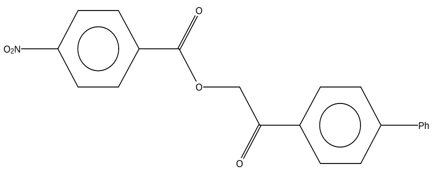

JIVJOQ

Reference: Huey Chong Kwong, C.S.Chidan Kumar, Siau Hui Mah, Tze Shyang Chia, Ching Kheng Quah, Zi Han Loh, S.Chandraju, Gin Keat Lim (2017) *Plos One* ,**12,**e0170117

Formula: $C_{21}\ H_{15}\ N_1\ O_5$

Compound Name: 2-(biphenyl-4-yl)-2-oxoethyl 2-nitrobenzoate

Space Group:	Pna21	***Cell:***	***a*** 9.844(1)	***b*** 32.312(5)	***c*** 5.368(0)
Space Group No.:	33	***(Å,°)***	α 90.00	β 90.00	γ 90.00

R-Factor (%): 5.54 ***Temperature(K):*** 100 ***Density(g/cm³):*** 1.406

Parameters

Fragment 1
ANG1 (A) 11.771
ANG2 (A) 58.652
ANG3 (A) 61.685

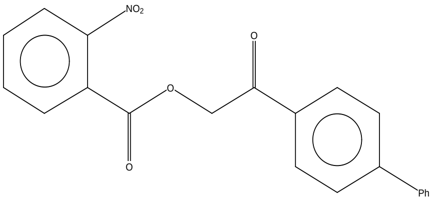

CIYCAQ

Reference: C.S.C.Kumar, Tze Shyang Chia, S.Chandraju, Chin Wei Ooi, Ching Kheng Quah, Hoong-Kun Fun (2014) *Z.Krist.Cryst.Mater.* ,**229,**328

Formula: $C_{15}\ H_{10}\ Br_1\ N_1\ O_5$

Compound Name: 2-(4-Bromophenyl)-2-oxoethyl 2-nitrobenzoate

Space Group:	P21/c	***Cell:***	***a*** 15.596(1)	***b*** 25.103(3)	***c*** 7.254(0)
Space Group No.:	14	***(Å,°)***	α 90.00	β 99.44(0)	γ 90.00

R-Factor (%): 3.83 ***Temperature(K):*** 100 ***Density(g/cm³):*** 1.727

Parameters

Fragment 1
ANG1 (A) 24.998
ANG2 (A) 57.508
ANG3 (A) 65.709

Fragment 2
ANG1 (A) 69.517
ANG2 (A) 40.068
ANG3 (A) 89.505

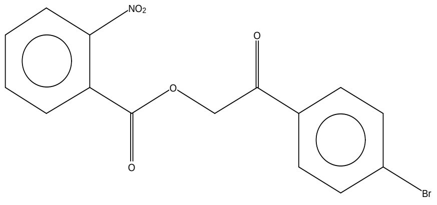

Search: search2 (Sun Oct 13 16:31:09 2019): Hits 5-7

CIYCEU

Reference: C.S.C.Kumar, Tze Shyang Chia, S.Chandraju, Chin Wei Ooi, Ching Kheng Quah, Hoong-Kun Fun (2014) *Z.Krist.Cryst.Mater.* ,**229,**328

Formula: C_{15} H_{10} Br_1 N_1 O_5

Compound Name: 2-(4-Bromophenyl)-2-oxoethyl 3-nitrobenzoate

Space Group:	P-1	***Cell:***	***a*** 9.311(0)	***b*** 12.098(1)	***c*** 14.175(1)
Space Group No.:	2	***(Å,°)***	α 100.61(0)	β 100.35(0)	γ 105.99(0)

R-Factor (%): 5.48 ***Temperature(K):*** 296 ***Density(g/cm^3):*** 1.653

Parameters

Fragment 1

**ANG1 (A)**	4.742
**ANG2 (A)**	1.620
**ANG3 (A)**	4.446

Fragment 2

**ANG1 (A)**	7.368
**ANG2 (A)**	3.781
**ANG3 (A)**	4.080

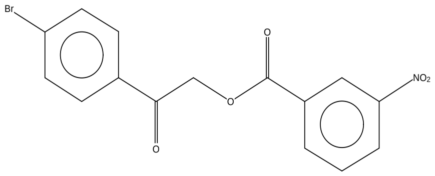

CIYCIY

Reference: C.S.C.Kumar, Tze Shyang Chia, S.Chandraju, Chin Wei Ooi, Ching Kheng Quah, Hoong-Kun Fun (2014) *Z.Krist.Cryst.Mater.* ,**229,**328

Formula: C_{15} H_{10} Br_1 N_1 O_5

Compound Name: 2-(4-Bromophenyl)-2-oxoethyl 4-nitrobenzoate

Space Group:	P21/c	***Cell:***	***a*** 14.896(0)	***b*** 12.707(0)	***c*** 7.371(0)
Space Group No.:	14	***(Å,°)***	α 90.00	β 96.20(0)	γ 90.00

R-Factor (%): 4.45 ***Temperature(K):*** 100 ***Density(g/cm^3):*** 1.744

Parameters

Fragment 1

**ANG1 (A)**	82.031
**ANG2 (A)**	38.088
**ANG3 (A)**	87.661

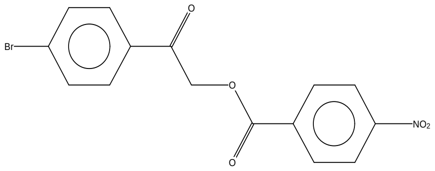

PODRAD

Reference: C.S.Chidan Kumar, C.Yohannan Panicker, Hoong-Kun Fun, Y.Sheena Mary, B.Harikumar, S.Chandraju, Ching Kheng Quah, Chin Wei Ooi (2014) *Spectrochim.Acta,Part A* , **126,**208

Formula: C_{15} H_{10} Cl_1 N_1 O_5

Compound Name: 2-(4-chlorophenyl)-2-oxoethyl 3-nitrobenzoate

Space Group:	P-1	***Cell:***	***a*** 9.258(1)	***b*** 12.106(1)	***c*** 14.123(1)
Space Group No.:	2	***(Å,°)***	α 100.79(0)	β 100.20(1)	γ 106.21(0)

R-Factor (%): 5.60 ***Temperature(K):*** 297 ***Density(g/cm^3):*** 1.466

Parameters

Fragment 1

**ANG1 (A)**	3.702
**ANG2 (A)**	2.438
**ANG3 (A)**	3.207

Fragment 2

**ANG1 (A)**	5.918
**ANG2 (A)**	3.974
**ANG3 (A)**	2.999

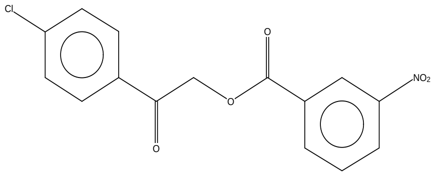

Supplement: Supplementary file 5 [file e-75-01719-sup4.pdf]
